# Engineered skeletal muscle recapitulates human muscle development, regeneration and dystrophy

**DOI:** 10.1002/jcsm.13094

**Published:** 2022-10-18

**Authors:** Mina Shahriyari, Md Rezaul Islam, Sadman M. Sakib, Malte Rinn, Anastasia Rika, Dennis Krüger, Lalit Kaurani, Verena Gisa, Mandy Winterhoff, Harithaa Anandakumar, Orr Shomroni, Matthias Schmidt, Gabriela Salinas, Andreas Unger, Wolfgang A. Linke, Jana Zschüntzsch, Jens Schmidt, Rhonda Bassel‐Duby, Eric N. Olson, André Fischer, Wolfram‐Hubertus Zimmermann, Malte Tiburcy

**Affiliations:** ^1^ Institute of Pharmacology and Toxicology University Medical Center Göttingen, Georg August University Göttingen Germany; ^2^ DZHK (German Centre for Cardiovascular Research), partner site Göttingen Göttingen Germany; ^3^ Department for Epigenetics and Systems Medicine in Neurodegenerative Diseases German Center for Neurodegenerative Diseases (DZNE) Göttingen Göttingen Germany; ^4^ NGS Integrative Genomics Core Unit, Institute of Human Genetics University Medical Center Göttingen, Georg August University Göttingen Germany; ^5^ Department of Neurology, Neuromuscular Center University Medical Center Göttingen, Georg August University Göttingen Germany; ^6^ Institute of Physiology II University of Münster Münster Germany; ^7^ Department of Neurology and Pain Treatment, Immanuel Klinik Rüdersdorf University Hospital of the Brandenburg Medical School Theodor Fontane Rüdersdorf bei Berlin Germany; ^8^ Faculty of Health Sciences Brandenburg Brandenburg Medical School Theodor Fontane Rüdersdorf bei Berlin Germany; ^9^ Department of Molecular Biology University of Texas Southwestern Medical Center Dallas TX USA; ^10^ Senator Paul D. Wellstone Muscular Dystrophy Cooperative Research Center University of Texas Southwestern Medical Center Dallas TX USA; ^11^ Hamon Center for Regenerative Science and Medicine University of Texas Southwestern Medical Center Dallas TX USA; ^12^ Cluster of Excellence ‘Multiscale Bioimaging: from Molecular Machines to Networks of Excitable Cells’ (MBExC) University of Göttingen Göttingen Germany; ^13^ Fraunhofer Institute for Translational Medicine and Pharmacology (ITMP) Göttingen Germany

**Keywords:** Duchenne muscular dystrophy, hypaxial dermomyotome, limb muscle, satellite cells, skeletal muscle organoid, somite, tissue engineering

## Abstract

**Background:**

Human pluripotent stem cell‐derived muscle models show great potential for translational research. Here, we describe developmentally inspired methods for the derivation of skeletal muscle cells and their utility in skeletal muscle tissue engineering with the aim to model skeletal muscle regeneration and dystrophy in vitro.

**Methods:**

Key steps include the directed differentiation of human pluripotent stem cells to embryonic muscle progenitors followed by primary and secondary foetal myogenesis into three‐dimensional muscle. To simulate Duchenne muscular dystrophy (DMD), a patient‐specific induced pluripotent stem cell line was compared to a CRISPR/Cas9‐edited isogenic control line.

**Results:**

The established skeletal muscle differentiation protocol robustly and faithfully recapitulates critical steps of embryonic myogenesis in two‐dimensional and three‐dimensional cultures, resulting in functional human skeletal muscle organoids (SMOs) and engineered skeletal muscles (ESMs) with a regeneration‐competent satellite‐like cell pool. Tissue‐engineered muscle exhibits organotypic maturation and function (up to 5.7 ± 0.5 mN tetanic twitch tension at 100 Hz in ESM). Contractile performance could be further enhanced by timed thyroid hormone treatment, increasing the speed of contraction (time to peak contraction) as well as relaxation (time to 50% relaxation) of single twitches from 107 ± 2 to 75 ± 4 ms (*P* < 0.05) and from 146 ± 6 to 100 ± 6 ms (*P* < 0.05), respectively. Satellite‐like cells could be documented as largely quiescent PAX7^+^ cells (75 ± 6% Ki67^−^) located adjacent to muscle fibres confined under a laminin‐containing basal membrane. Activation of the engineered satellite‐like cell niche was documented in a cardiotoxin injury model with marked recovery of contractility to 57 ± 8% of the pre‐injury force 21 days post‐injury (*P* < 0.05 compared to Day 2 post‐injury), which was completely blocked by preceding irradiation. Absence of dystrophin in DMD ESM caused a marked reduction of contractile force (−35 ± 7%, *P* < 0.05) and impaired expression of fast myosin isoforms resulting in prolonged contraction (175 ± 14 ms, *P* < 0.05 vs. gene‐edited control) and relaxation (238 ± 22 ms, *P* < 0.05 vs. gene‐edited control) times. Restoration of dystrophin levels by gene editing rescued the DMD phenotype in ESM.

**Conclusions:**

We introduce human muscle models with canonical properties of bona fide skeletal muscle in vivo to study muscle development, maturation, disease and repair.

## Introduction

Pluripotent stem cell (PSC)‐derived organotypic cultures with structural and functional properties of native human tissue are increasingly utilized for disease modelling and drug screening applications. Organotypic skeletal muscle cultures are highly sought after, because of the central role of skeletal muscle in disease (e.g., myopathies) and drug effects (e.g., insulin). Early studies have demonstrated that it only requires MyoD overexpression in fibroblasts to activate myogenic programmes.^1^ Muscle stem cells can also be isolated from muscle biopsies, but rapidly lose their stem cell properties with expansion often requiring immortalization to provide consistent cell quantity and quality.^2^, ^3^ Derivation of skeletal muscle cells from human PSC (hPSC) can in principle overcome this limitation and combined with tissue/organoid engineering methods may allow for an emulation of embryonic myogenesis from a cell to tissue level.

The derivation of skeletal muscle cells from PSC has been demonstrated previously by either transfection or transduction of myogenic transgenes^4^, ^5^, ^6^, ^7^, ^8^, ^9^, ^10^ or directed, transgene‐free differentiation under controlled growth factors or small‐molecule stimulation.^11^, ^12^, ^13^, ^14^, ^15^, ^16^, ^17^ Recently, more advanced neuromuscular organoids have been introduced that recapitulate characteristic steps of embryonic neuromuscular co‐development.^18^, ^19^ These innovative cell models have shown potential for preclinical modelling of human neuromuscular disease, such as laminopathies,^20^, ^21^ Duchenne muscular dystrophy (DMD)^14^, ^21^, ^22^, ^23^, ^24^ and myasthenia gravis.^18^, ^25^

Several studies have applied tissue engineering methods to generate skeletal muscle from hPSC‐derived cells in vitro,^6^, ^21^, ^26^ collectively suggesting a potential of 3D skeletal muscle for disease modelling and regenerative medicine. However, the functional output of in vitro muscle is still far from postnatal muscle even though improvements have been demonstrated using a specific cell culture supplement.^26^ For the only transgene‐free model published so far, muscle function was not reported.^21^ In a previous study using rat primary myocytes, our group demonstrated that the application of collagen/Matrigel® hydrogels in combination with isometric loading generates engineered skeletal muscle (ESM) with physiological function and a regenerative satellite cell niche in vitro.^27^

Here, we report a transgene‐free and serum‐free human muscle protocol that closely follows developmental trajectories to induce somitogenesis and muscle formation in 2D and 3D. Human ESMs respond to developmentally relevant cues, such as triiodothyronine, with advanced maturation, demonstrating physiological growth potential, and display functional regeneration by satellite‐like cells in vitro. In addition, ESMs recapitulate the contractile deficit of DMD and its rescue by CRISPR/Cas9 myoediting.

## Methods

### Generation of skeletal muscle from human pluripotent stem cells

Human PSC lines (please see Methods in the supporting information for details) were maintained on 1:120 Matrigel™ (BD) in phosphate‐buffered saline (Thermo Fisher Scientific)‐coated plates and cultured in StemMACS iPS‐Brew XF (Miltenyi Biotec) at 37°C and 5% CO_2_.

For skeletal muscle differentiation in 2D, 1.3 × 10^4^ to 2.1 × 10^4^ cells/cm^2^ PSC were seeded in iPS‐Brew XF medium with 5 μmol/L of Y27632 (Stemgent) for 24 h. iPS‐Brew XF medium was then replaced with daily refreshed N2‐CLF medium for 4 days. N2‐CLF medium consisted of DMEM (Thermo Fisher Scientific) with 1% Pen/Strep, 1% N‐2 Supplement and 1% MEM non‐essential amino acid solution (N2 basal medium, all Thermo Fisher Scientific), 10 μmol/L CHIR99021 (Stemgent), 0.5 μmol/L LDN193189 (Stemgent) and 10 ng/mL FGF‐2 (PeproTech). At Day 4, the medium was exchanged with N2‐FD medium every 24 h until Day 6. N2‐FD medium contained N2 basal medium, 20 ng/mL FGF‐2 and 10 μmol/L DAPT (Tocris). For Days 6 and 7, the medium was replaced with N2‐FDH (N2 basal medium, 20 ng/mL FG8F‐2, 10 μmol/L DAPT and 10 ng/mL HGF [PeproTech]). The medium was switched to N2‐DHK medium on Days 8, 9, 10 and 11 (N2 basal medium, 10 μmol/L DAPT, 10 ng/mL HGF and 10% knockout serum replacement [Thermo Fisher Scientific]). From Days 13 to 22, myogenic cells were cultured in expansion medium (N2 basal medium, 10% knockout serum replacement and 10 ng/mL HGF). To further differentiate the cells in monolayer culture, Day 22 skeletal myocytes were enzymatically dissociated with TrypLE (Thermo Fisher Scientific) for 5 to 7 min at 37°C and replated on 1:120 Matrigel™‐coated plates at a density of 60 000–70 000 cells/cm^2^ in expansion medium with 5 μmol/L Y27632 (Stemgent). Skeletal myocyte maturation was done in DMEM with 1% Pen/Strep, 1% N‐2 Supplement and 2% B‐27 Supplement (maturation medium).

To generate SMOs in 3D, 0.8 × 10^6^ PSCs resuspended in 157.5 μL of StemMACS iPS‐Brew XF medium with 5 μmol/L Y27632, 10 ng/mL FGF‐2 and 10% knockout serum replacement were cast into a hydrogel as a 250 μL volume reconstitution mixture comprised of (i) 36 μL of 6.5 mg/mL (final amount of 0.23 mg) acid soluble collagen type 1 (LLC Collagen Solutions), (ii) 36 μL of concentrated 2× DMEM (Thermo Fisher Scientific) serum‐free medium, (iii) 6.75 μL of NaOH 0.1 N (Carl Roth) and (iv) 10% v/v Matrigel™ (BD). PSCs in hydrogel were then differentiated as outlined above for 2D cells.

To make ESM, 1.25 × 10^6^ of Day 22 PSC‐derived skeletal myocytes, which were resuspended in 157.5 μL of expansion medium with 5 μmol/L Y2763, were cast into a hydrogel with a final 250 μL per ESM volume mixture of (i) 36 μL of 6.5 mg/mL (final amount of 0.23 mg) acid soluble collagen type 1, (ii) 36 μL of concentrated 2× DMEM medium, (iii) 6.75 μL of NaOH 0.1 N and (iv) 10% v/v Matrigel™ (please see Methods in the supporting information for details).

### Statistical analysis

Data were analysed using GraphPad Prism 7 software and presented as mean ± SEM. Statistical analyses were done using unpaired, two‐tailed, Student's *t* test and one‐way or two‐way ANOVA with specified multiple comparison tests where appropriate (please refer to figure legends). *P* < 0.05 was considered significant. *n* indicates the number of biological replicates.

Please see Methods in the supporting information for further experimental details.

## Results

### Embryonic myogenesis by directed differentiation of human pluripotent stem cells in 2D and 3D

**Figure 1:**
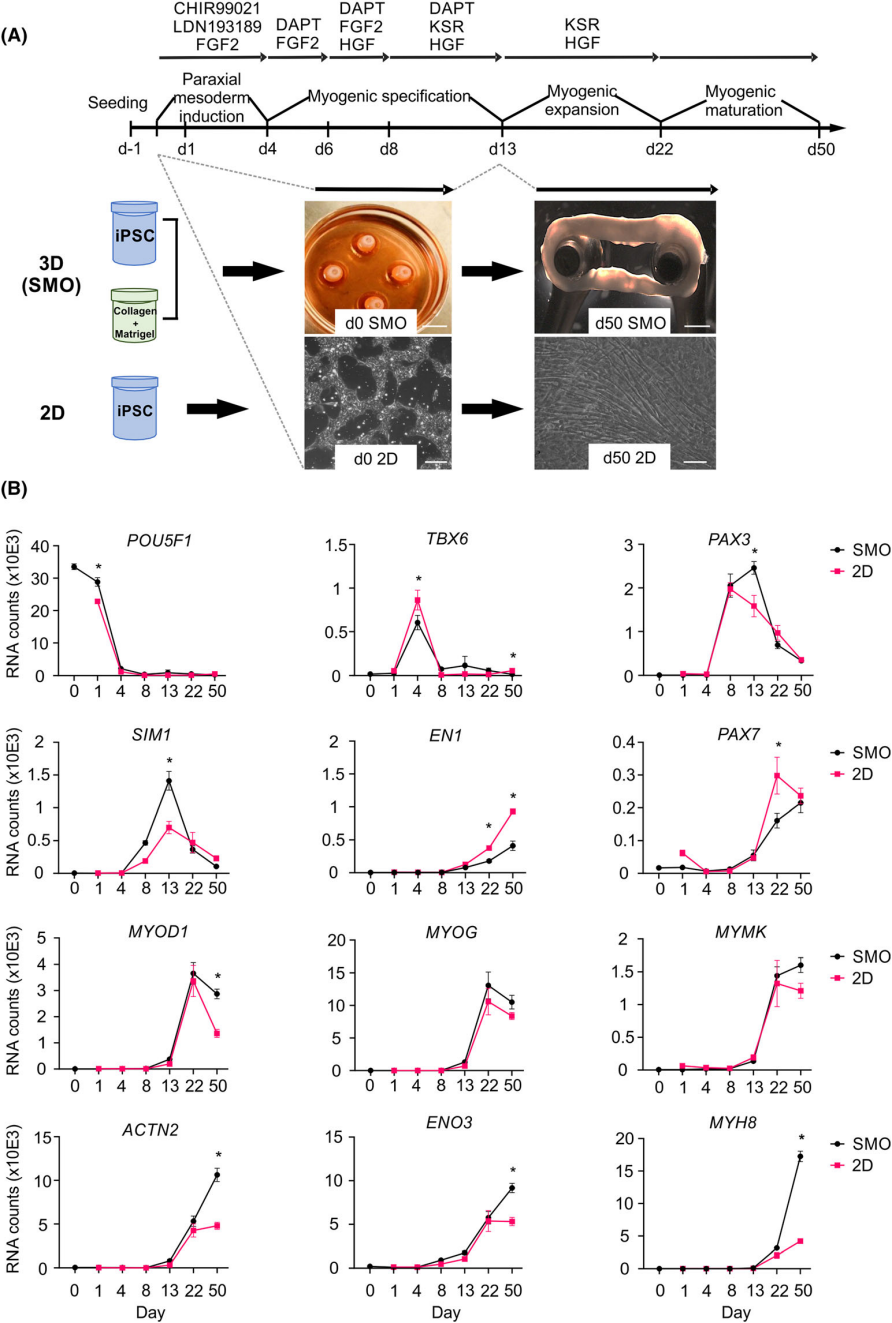
Skeletal myocyte differentiation from human pluripotent stem cells (PSCs) in 2D and 3D cultures. (A) Summary of the protocol for directed skeletal muscle differentiation from PSCs indicating the sequence and the timing of factor addition to modulate specific signalling pathways involved in skeletal myogenesis. Skeletal muscle organoids (SMOs) were generated from induced PSC (iPSC) mixed with collagen type 1 and Matrigel™ in a ring‐shaped hydrogel. After consolidation in PDMS casting moulds, SMOs were directed towards skeletal muscle using the indicated protocol established in 2D monolayer cultures. Scale bars: 5 and 1 mm (3D panels); 50 μm (2D panels). (B) Transcript levels (RNA counts measured by nCounter) of signature genes for pluripotency (*POU5F1*), paraxial mesoderm (*TBX6*), somitogenesis (*PAX3*, *SIM1* and *EN1*), myogenic transcription factors (*PAX7*, *MYOD1* and *MYOG*), structural assembly (*MYMK* and *ACTN2*) and secondary myogenesis (*ENO3* and *MYH8*) during skeletal muscle differentiation from human PSCs in SMO and 2D; *n* = 3–5 per time point and group, ^*^ *P* < 0.05 by two‐way analysis of variance (ANOVA) and Sidak's multiple comparison test

To robustly generate human skeletal muscle, we adapted several principles previously identified to be crucial for directed skeletal muscle differentiation (final optimized protocol in *Figure* 1A).^12^, ^14^, ^17^, ^28^ As 2D cultures of skeletal myocytes do not develop the spatial and structural organization of skeletal muscle in vivo,^25^ we also investigated if muscle differentiation could be recapitulated in a 3D format. To test this, we embedded undifferentiated induced (iPSC) in a collagen/Matrigel hydrogel to obtain SMOs (*Figure* 1A). We first asked if the temporal sequence of muscle cell development was comparable to parallel 2D differentiation. RNA expression analyses showed a similar decrease in pluripotency gene (*POU5F1*) and increase of paraxial mesoderm gene (*TBX6*) expression by a modification of BMP (inhibition) and Wnt (activation) signalling with 0.5 μmol/L LDN 193189 and 10 μmol/L CHIR99021.^11^, ^12^ Following paraxial mesoderm induction, the expression of *PAX3* and *SIM1* in the presence of FGF2, HGF^12^ and Notch 1 inhibition with DAPT (10 μmol/L; γ‐secretase inhibition^14^) was an indication of somitogenesis with an enhanced expression of dermomyotomal progenitor cell transcripts (*PAX3* and *SIM1*) in SMO. Interestingly, low expression of *EN1* at the peak of *SIM1* expression (Day 13) may indicate a predominantly hypaxial dermomyotomal progenitor pattern.^29^ This was followed by a largely comparable emergence of muscle progenitor transcripts *PAX7*, *MYOD1*, *MYOG* and *MYMK*; *MYOD1* expression remained more abundant at Day 50 in SMO. In addition, robust increases in *ACTN2*, *ENO3* and *MYH8* expression suggested emergence of myocytes and secondary myogenesis between Days 22 and 52, which was enhanced in SMO (*Figure* 1B). The temporal transcriptome changes were corroborated by immunostaining demonstrating the loss of OCT4, appearance of muscle progenitor populations expressing PAX3 and LBX1 at Days 8 and 13 followed by consecutive appearance of PAX7^+^, MYOD1^+^, MYOG^+^ and ACTN2^+^ myocytes in 2D and SMO cultures (*Figure* S1).

**Figure 2:**
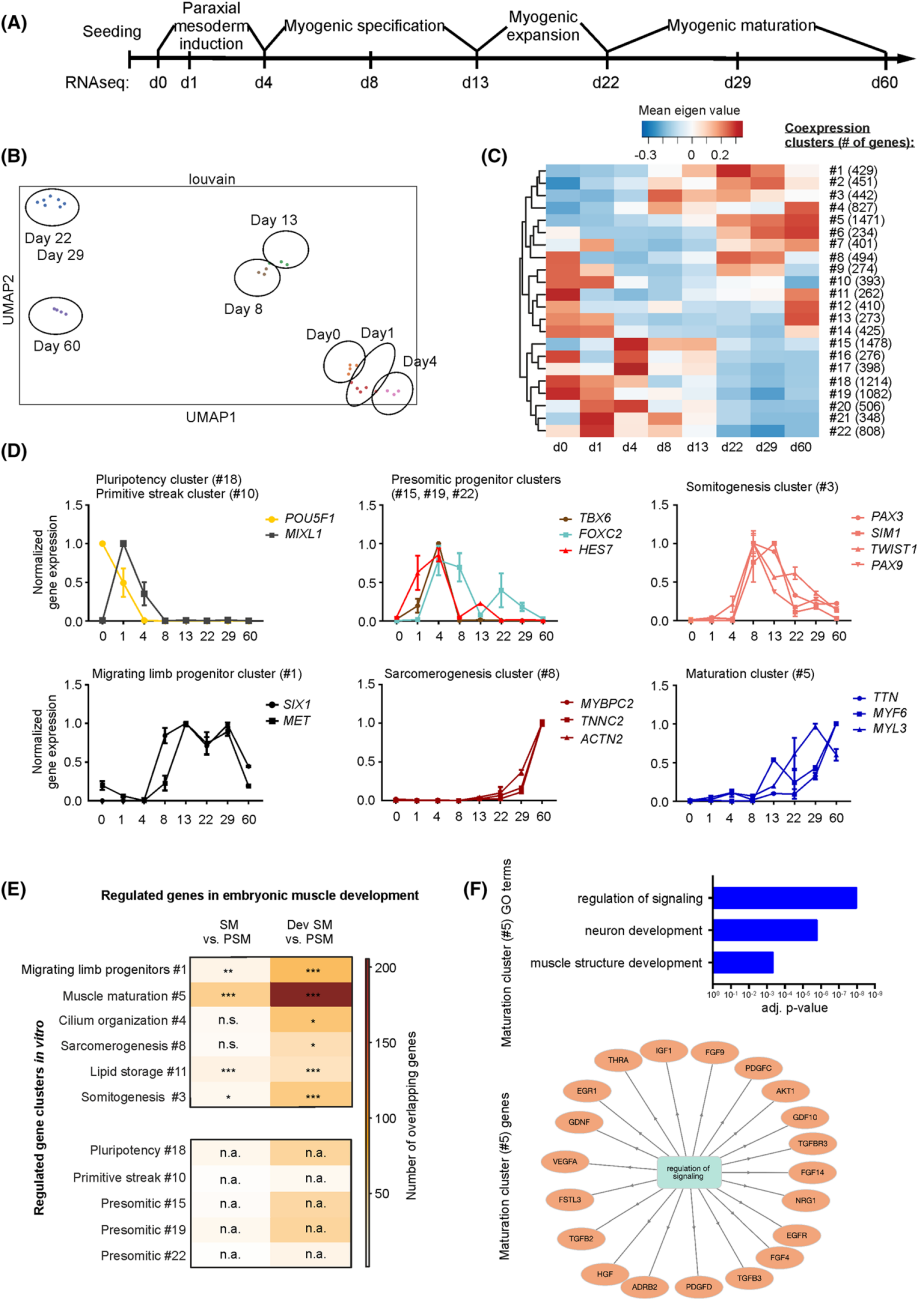
Developmental transcriptome patterns in pluripotent stem cell (PSC) skeletal myocyte differentiation. (A) Scheme of skeletal muscle differentiation from human PSCs (HES2) with sampling time points for RNA sequencing. (B) Unsupervised clustering of the samples from different time points. (C) Weighted co‐expression analysis identified 22 clusters of genes with similar expression dynamics (co‐expression clusters); a heatmap of mean eigenvalues is displayed. (D) Normalized expression levels (reads per kilobase million [RPKM]) of indicated signature genes in identified co‐expression clusters, *n* = 2–4 per time point. (E) Developmentally regulated genes were identified based on a published human embryonic muscle data set.^13^ The table indicates the overlap of co‐expression cluster genes to genes regulated between presomitic mesoderm (PSM) and nascent somite (SM) or presomitic mesoderm (PSM) and developed somite (Dev SM). Overlap is graded as either not significant (n.s.), ^*^ *P* < 0.05, ^**^ *P* < 0.01 or ^***^ *P* < 0.001 by Fisher's exact test. The colour codes for the number of overlapping genes. As differentially expressed genes were obtained by comparing to SM and Dev SM to PSM, preceding developmental processes (i.e., paraxial mesoderm formation and earlier) are not represented in the embryo data set and therefore cannot overlap with in vitro processes (labelled as not applicable, n.a.). Clusters that were not muscle related and did not significantly overlap were omitted. (F) Gene Ontology (GO) terms specifically enriched in co‐expression cluster #5 (top panel). List of genes associated with ‘regulation of signaling’ in co‐expression cluster #5 (bottom panel)

To obtain more insight into the global developmental patterns of the skeletal muscle differentiation protocol in vitro, we subjected RNAseq data obtained at selected time points in 2D differentiation to bioinformatic analyses (*Figure* 2A). Unbiased clustering separated the distinct time points into mesoderm induction (Days 0, 1 and 4), myogenic specification (Days 8 and 13), early (Days 22 and 29) and advanced (Day 60) myogenic maturation (*Figure* 2B). Further clustering the genes by weighted co‐expression analysis identified 22 gene clusters with remarkable overlap to the biological processes of skeletal muscle differentiation characterized by the expression of developmental signature genes reflecting primitive streak formation (cluster #10), paraxial mesoderm formation and patterning (clusters #15, 19 and 22), formation of dorsal (dermomyotome) and ventral somite (sclerotome, cluster #3), migrating limb progenitors (cluster #1), sarcomere formation (cluster #8) and muscle maturation (cluster #5; *Figure* 2C,D and *Table* S1).

We next asked if the identified gene clusters overlap with developmentally regulated genes of human embryonic muscle making use of a published data set (GSE90876^13^). Interestingly, several of the bioinformatically identified clusters from skeletal myogenesis in vitro showed significant overlap with embryonic development in vivo (*Figure* 2E). We then utilized the co‐expression analysis to dissect processes coinciding with muscle maturation (cluster #5) to extract information to support muscle maturation in vitro. Interestingly, cluster #5 was highly enriched in signalling transcripts (*Figure* 2F). Among them, we identified several signalling pathways that have been associated with muscle maturation such as NRG1, IGF1/VEGF and thyroid hormone indicating that our protocol emulates central mechanisms of muscle development in vivo. In summary, we demonstrate the successful recapitulation of key developmental stages of human embryonic muscle development in 2D and in a 3D organoid approach (SMO).

### Generation of skeletal muscle with organotypic function

**Figure 3:**
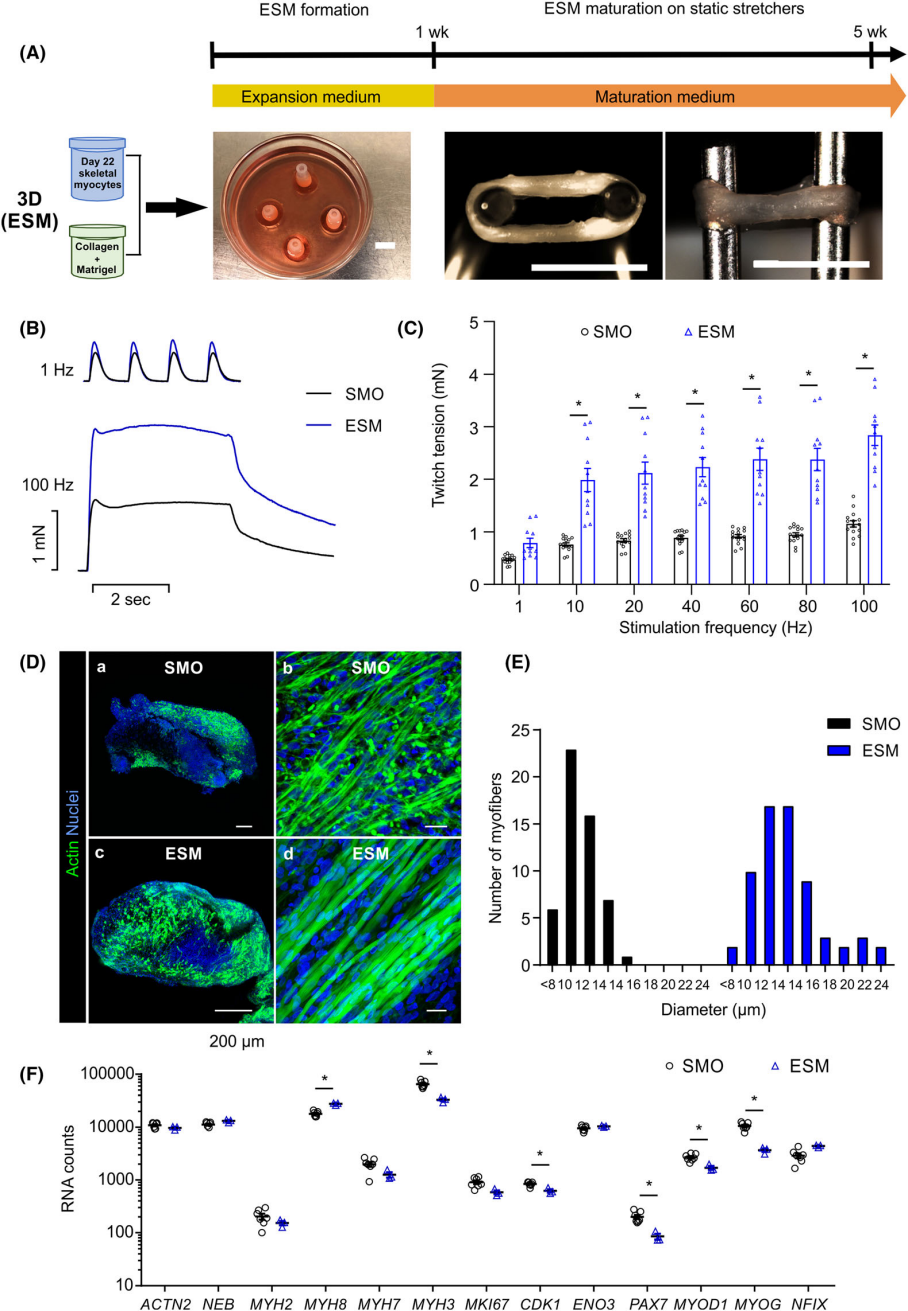
Advanced development of skeletal muscle function in human engineered skeletal muscle (ESM). (A) Scheme of ESM generation from human PSC‐derived skeletal myocytes with collagen type 1 and Matrigel™ in a ring‐shaped hydrogel. ESM formation in expansion medium for 1 week in PDMS casting moulds, functional maturation under isometric mechanical load (ESM on metal hooks). Scale bar: 5 mm. (B) Representative original recordings of single twitches at 1 Hz and tetanic contraction at 100 Hz of skeletal muscle organoid (SMO) (black lines) and ESM (blue lines). (C) Twitch tension in response to increasing stimulation frequencies of SMO (black bars) and ESM (blue bars) after 4 weeks of maturation; *n* = 15 for SMO and *n* = 11 for ESM, ^*^ *P* < 0.05 by two‐way analysis of variance (ANOVA) and Tukey's multiple comparison test. (D) Immunostaining of ACTIN^+^ muscle cells (green) in cross sections and longitudinal sections of SMO and ESM after 4 weeks of maturation. Scale bar: 500 μm (A, C) and 20 μm (B, D). (E) Myofibre diameter distribution in SMO and ESM after 4 weeks of maturation. (F) Transcript levels (RNA counts measured by nCounter) of indicated muscle genes in SMO and ESM, *n* = 7/3 (SMO/ESM), ^*^ *P* < 0.05 by two‐way ANOVA and Sidak's multiple comparison test

As both 2D myocytes (*Video S1*) and SMO cultures (*Video S2*) demonstrated contractile activity, we further investigated if skeletal muscle tissue with skeletal muscle‐specific function can be generated from hPSC. Although SMOs recapitulate the embryonic muscle development in 3D, we tested the controlled assembly of differentiated PSC‐derived skeletal myocyte populations obtained from 2D directed differentiation into ESM as an alternative approach. Day 22 myocytes (identified as optimal time point based on palpable expression of MRFs and ACTN2, *Figure* 1B) were dissociated and allowed to self‐organize after suspension in a collagen/Matrigel^TM^ hydrogel and transfer into a circular casting mould (*Figure* 3A). Immunostaining of the Day 22 input cell populations revealed a cell composition consisting of 43 ± 4% PAX7^+^, 52 ± 2% MYOD1^+^ and 49 ± 4% MYOGENIN^+^ (*n* = 9–13 differentiations; *Figure* S2A). Flow cytometry showed comparable efficiency for one human embryonic stem cell (HESC) and four different iPSC lines, supporting the robustness and reproducibility of the protocol (*Figure* S2B). After formation of a compact tissue ring (after 4–7 days), ESMs were transferred to metal holders for further maturation (*Figure* 3A). After 1–2 weeks, spontaneous contractions were observed in ESM (*Video S3*).

SMOs and ESMs demonstrated robust force generation after 4 weeks of maturation. At 1 Hz electrical stimulation, SMOs and ESMs generated single twitches, whereas at higher frequencies, tetanic contractions were observed, which were maximal at 100 Hz and significantly stronger in ESM (2.8 ± 0.2 vs. 1.2 ± 0.1 mN in SMO, *n* = 11/15; *Figure* 3B,C). The cross‐sectional area populated with muscle cells tended to be lower in SMO compared to ESM (48 ± 3% vs. 58 ± 3% [*n* = 3]). In addition, the diameter of the myofibres was significantly smaller in SMO compared to ESM, likely explaining the differences in function (*Figure* 3D,E). The difference in maturation was further supported by higher expression of embryonic myosin (*MYH3*), lower expression of perinatal myosin (*MYH8*) and higher expression of cell cycle (*CDK1*) and muscle progenitor transcripts (*PAX7*, *MYOD1* and *MYOG*) in SMO compared to ESM (*Figure* 3F). Collectively, both tissue engineering approaches (SMO and ESM) yield skeletal muscle with physiological function, with enhanced skeletal muscle maturation in ESM.

Next, we were interested if the ESM protocol is in principle suitable to generate skeletal muscle from human primary skeletal myocytes (pSkMs) and how it compares to PSC‐derived ESM. Although force‐generating tissue was uniformly generated, we noticed lower force development (0.4 ± 0.1 vs. 2 ± 0.3 mN, *n* = 6/3 in pSkM ESM vs. PSC ESM) and high functional inter‐patient variability in pSkM ESM (*Figure* S3A,B). As muscle‐related genes were mostly more abundant in pSkM ESM (*Figure* S3C), we hypothesized that the lack of mesenchymal support cells may contribute to the lower function in pSkM ESM. To test this, we sorted mesenchymal cells (MCs) from PSC skeletal myocyte cultures and obtained an ~80% pure population of PDGFRA^+^ and FAP^+^ MC. Primary skeletal myocytes were then mixed with isolated PSC MC and compared to non‐MC‐supplemented ESM (*Figure* S3D). MC supplementation increased the twitch tension by 3.5 ± 0.1‐fold (*n* = 4, *P* < 0.05) indicating that the MC population supports muscle development (*Figure* S3E).

### Essential cellular heterogeneity as a result of directed myogenesis in vitro

**Figure 4:**
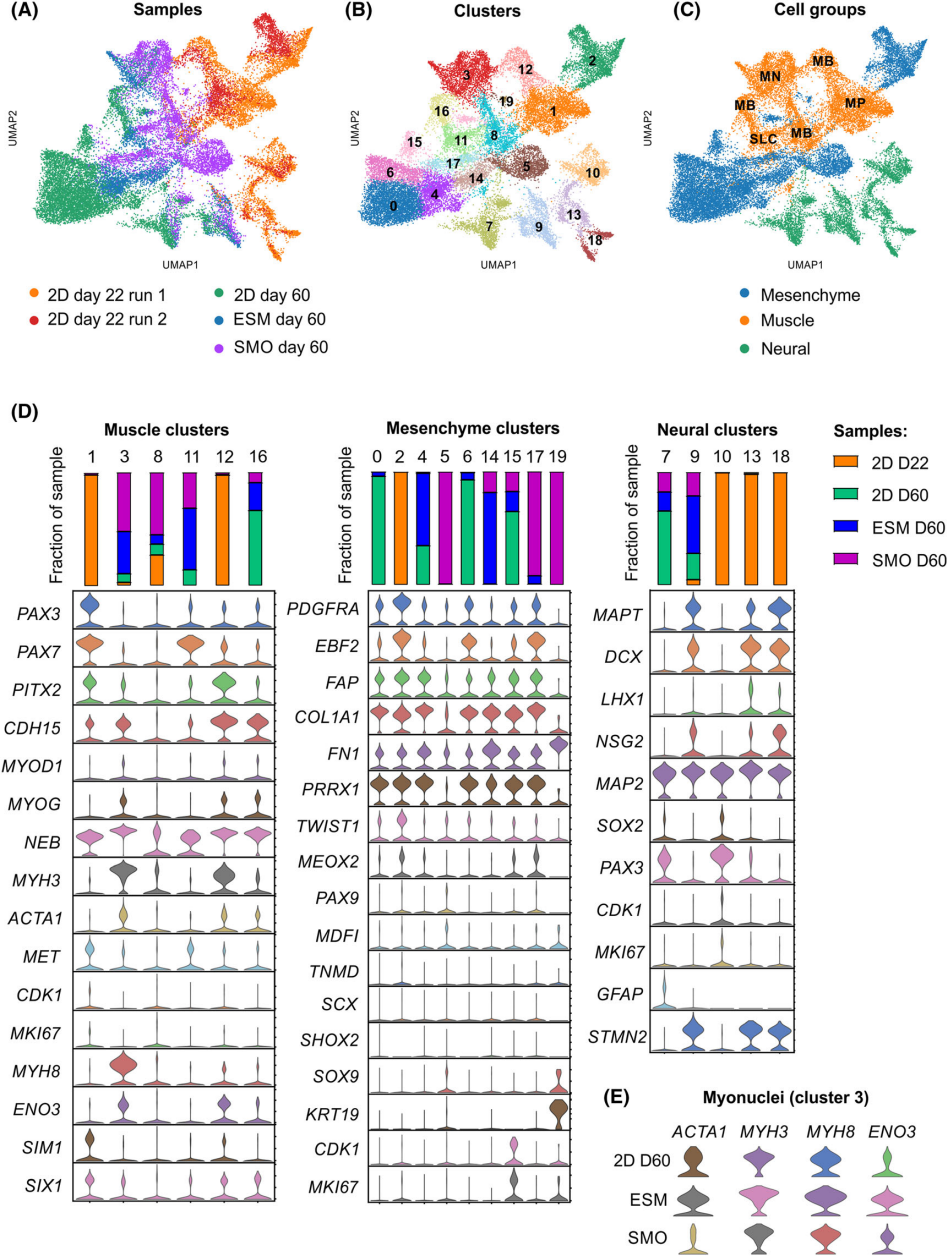
Cellular composition of differentiated skeletal myogenic cultures in 2D and 3D. (A) Unsupervised clustering (Uniform Manifold Approximation and Projection [UMAP]) of single‐nucleus transcriptomes. Colour coding indicates different input samples (2D Day 22 skeletal muscle cultures, 2D Day 60 skeletal muscle cultures, 3D Day 60 engineered skeletal muscle (ESM) and Day 60 skeletal muscle organoid (SMO). (B) Unsupervised clustering (UMAP) of single‐nucleus transcriptomes identifies 19 cell clusters. (C) Unsupervised clustering (UMAP) of single‐nucleus transcriptomes to identify major cell groups based on enrichment of muscle, neural and mesenchymal genes. Muscle clusters are further specified as myogenic progenitors (MP), myoblasts (MB), satellite‐like cell (SLC) and myonuclei (MN) based on transcriptional profiles indicated in (D). (D) Transcriptional profiles of the clusters separated by major cell groups (muscle, mesenchyme and neural). The relative contribution of the different samples to each cluster is indicated in the bar graphs. (E) Comparison of genes associated with secondary myogenesis and maturation in the myonuclei cluster (#3) between 2D Day 60, ESM Day 60 and SMO Day 60 samples.

Skeletal muscle differentiation in PSC yields heterogeneous cell populations, which may be beneficial for muscle formation in vitro as demonstrated for a sorted MC fraction (*Figure S3*). We performed single‐nucleus RNA sequencing to investigate whether our directed differentiation recapitulates development of the heterologous muscle cell populations found in bona fide skeletal muscle. We investigated Day 22 cultures as input cell population for ESM and compared it with later stage cultures in 3D (SMO and ESM after 4 weeks of maturation) or parallel 2D cultures (2D Day 60). The temporal difference in development between D22 and D60 cultures was clearly reflected by separation of the corresponding populations (*Figure* 4A). Importantly, the D22 cultures from two experimental runs (*n* = 9253 cells in total) were largely overlapping, supporting the robustness of the differentiation protocol in generating comparable cell populations (*Figure* 4A). Unsupervised clustering of all samples identified 19 different cell population of which clusters #1, 3, 8, 11, 12 and 16 were enriched in muscle genes, clusters #0, 2, 4, 5, 6, 14, 15, 17 and 19 were enriched in mesenchymal genes and clusters #7, 9, 10, 13 and 18 were enriched in neural genes (*Figure* 4B,C). Comparison of the contribution of each sample to the identified 19 clusters revealed clusters that were overrepresented in either 2D or 3D cultures. In line with the developmental trajectories, a myogenic progenitor (MP) population enriched in *PAX3* ^*+*^, *PAX7* ^*+*^, *SIM1* ^*+*^ and *MET* ^+^ cells was only identified in the D22 cultures (#1). A matured population of myonuclei (#3: *MYH8* ^*+*^, *ENO3* ^*+*^ and *ACTA1* ^*+*^) was only found in D60 cultures. Of note, higher expression levels and more frequent contribution to cluster #3 in 3D (SMO and ESM) versus 2D cultures support enhanced maturation in 3D (*Figure* 4D,E). Interestingly, cluster #11, which was also predominantly found in 3D cultures, was highly enriched in satellite cell markers (*PAX7*, *ITGA7*, *CALCR*, *EGFR* and *DLK1*) and inhibitors of cell cycle (*CDKN1C*) implicating a satellite‐like cell population (*Table* S2).

A mesenchymal population (#2) enriched in *PDGFRA* ^*+*^, *EBF2* ^*+*^, *FAP* ^*+*^, *MEOX2* ^*+*^ and *TWIST1* ^*+*^ cells was found in 2D Day 22 and similarly in SMO (#17) and 2D Day 60 (#6) cultures. In ESM, there was an apparent enrichment of MC populations (#4 and 14) with enhanced expression of ECM‐related genes (*COL1A1* and *FN1*). In SMO, two specific populations were identified. Cluster #5 showed persistent expression of sclerotome marker genes (*SOX9* and *MDFI*), whereas cluster #19 showed high expression of epidermal marker *KRT19*.

We also found evidence of neuronal co‐development during muscle differentiation. Day 22 cultures contained a PAX3^+^ neuronal progenitor population (#10), a developing LHX1^+^, DCX^+^ spinal cord neuron population (#13) and more matured NSG2^+^ and STMN2^+^ neurons (#18). Matured NSG2^+^ and STMN2^+^ neurons were still present in ESM (#9) and to a lower degree in 2D and SMO. These data indicate that the introduced differentiation protocol supports the generation of cell populations found in bona fide neuromuscular development.

### Advancing muscle function in engineered skeletal muscle

**Figure 5:**
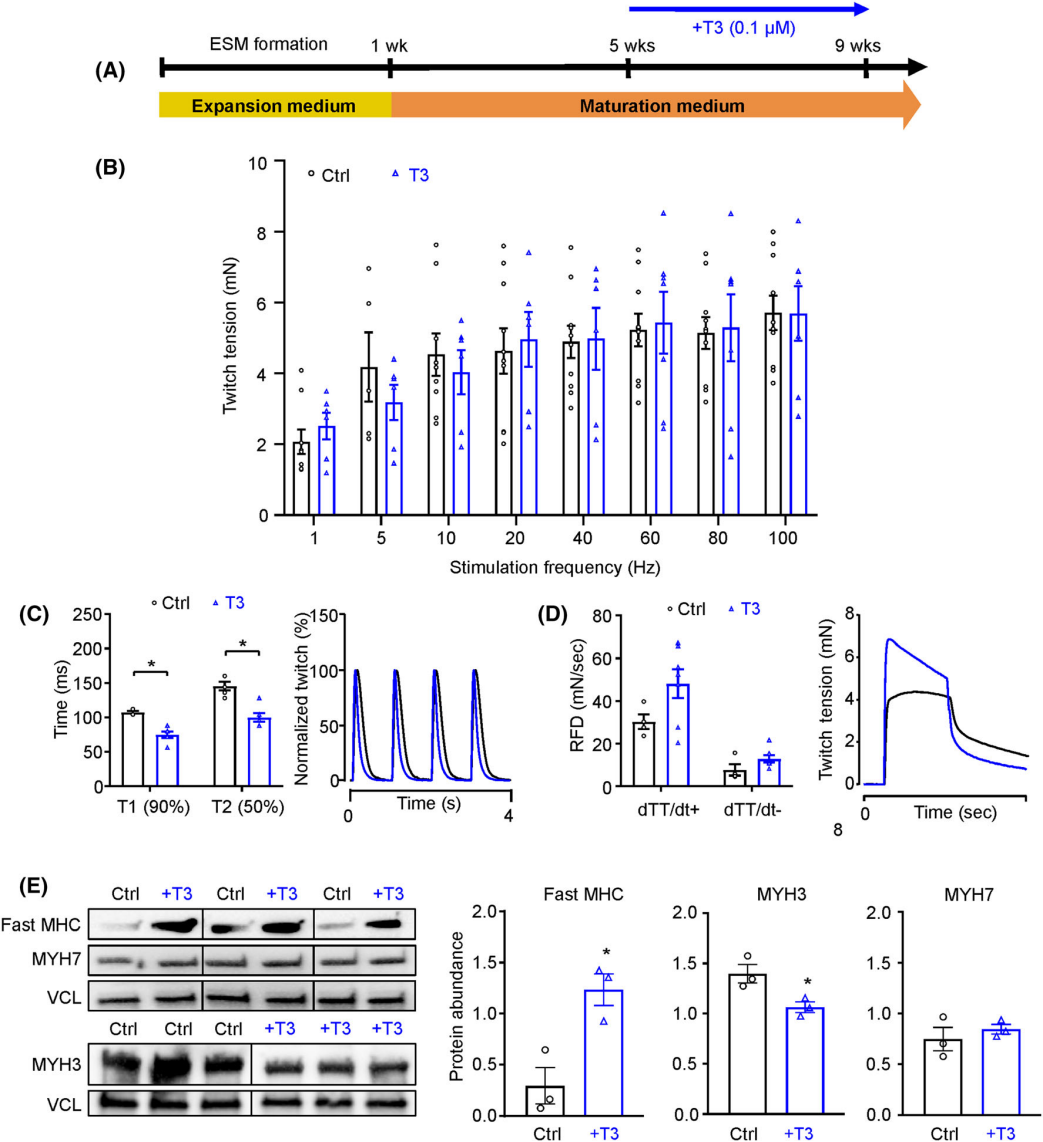
Advancing engineered skeletal muscle (ESM) function by thyroid hormone treatment. (A) Experimental design: ESM maturation for 9 weeks with or without additional application of 0.1 μmol/L triiodo‐l‐thyronine (T3) for 4 weeks. (B) Twitch tension in response to increasing stimulation frequencies of 9‐week‐old ESM cultured with (blue bars) or without T3 (black bars); *n* = 7–10 per group. (C) Quantification of contraction (T1) and relaxation (T2) time of single twitches of 9‐week‐old control (black bars) or +T3 (blue bars) ESM at 1 Hz (first panel); normalized representative traces of single twitches of 9‐week‐old control (black line) or +T3 (blue line) ESM at 1 Hz (second panel); *n* = 5–11 per group, ^*^ *P* < 0.05 by unpaired, two‐sided Student's *t* test. (D) Rate of force development (RFD; rate of contraction: dTT/dt+ and rate of relaxation: dTT/dt−) of 9‐week‐old control (black bars) or +T3 (blue bars) ESM at 100 Hz tetanus (first panel); representative traces of twitch tension of 9‐week‐old control (black line) or +T3 (blue line) ESM at 100 Hz tetanus (fourth panel); *n* = 4–11 per group, ^*^ *P* < 0.05 by unpaired, two‐tailed Student's *t* test. (E) Immunoblot for fast myosin heavy chain (MHC) isoforms, slow MHC (MYH7), embryonic MHC (MYH3) and loading control vinculin (VCL). Protein abundance of fast MHC (left panel), MYH7 (middle panel) and MYH3 (right panel) in 9‐week‐old ESM cultured with (blue bars) or without T3 (black bars); *n* = 3 per group, ^*^ *P* < 0.05 by unpaired, two‐tailed Student's *t* test

To further enhance ESM maturation and given the low expression of MYH2 (*Figure* 3F) as well as the finding of a high thyroid hormone receptor expression (maturation cluster; *Figure* 2F), we hypothesized that triiodo‐l‐thyronine (T3) addition may enhance the transition of myosin heavy chain isoform expression towards adult fast myosin isoforms and increase tetanic force production.^30^, ^31^ To test this hypothesis, we added T3 during maturation from Weeks 5 to 9 of ESM culture (*Figure* 5A).

T3 treatment did not influence the maximal twitch tension (*Figure* 5B), but clearly shortened the duration of single twitches of ESM. Accordingly, the speed of contraction (time to peak contraction—T1) of single twitches as well as relaxation (time to 50% relaxation—T2) at 9 weeks was significantly increased (*Figure* 5C). In addition, the rate of contraction and relaxation in tetanic contractions (100 Hz stimulation frequency) tended to be faster (*P* = 0.18) also in T3‐treated ESM (*Figure* 5D).

The tetanus threshold (i.e., frequency where single twitches fuse to tetani) is greater in mammalian adult fast muscle fibre in comparison to slow muscle fibres. The tetanus threshold of ESM with and without T3 treatment was analysed by calculation of a fusion index (*Figure* S4). T3 induced with a shift towards higher stimulation frequencies (50% fusion at 3.92 ± 0.24 vs. 5.44 ± 0.05 Hz in control ESM vs. ESM + T3, respectively, *n* = 8; *Figure* S4). Collectively, these functional data suggest that T3 enhances fast muscle properties of ESM. In line with the functional phenotype, we found that T3 treatment enhanced the abundance of mature fast myosin heavy chain (MHC) isoform, whereas the embryonic MYH3 isoform was reduced. The abundance of the slow myosin isoform MYH7 remained unchanged (*Figure* 5E). These molecular changes suggest that T3 supports maturation of fast skeletal muscle properties in ESM. The data also demonstrate that ESMs respond to physiological stimuli comparable to skeletal muscle in vivo.

**Figure 6:**
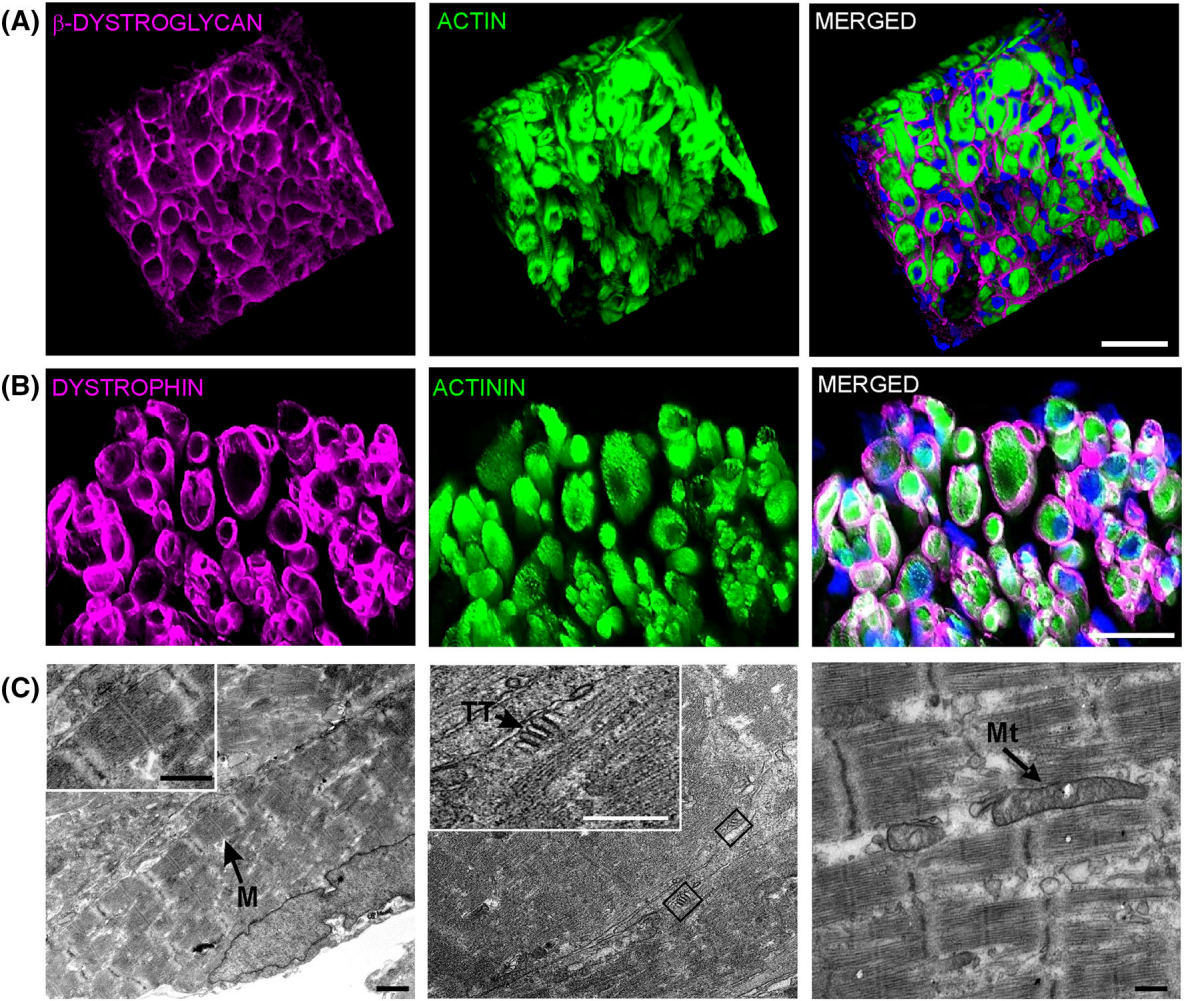
Maturation of muscle structure in engineered skeletal muscle (ESM). (A) Immunostaining of β‐dystroglycan (magenta) in the sarcolemma of actin^+^ muscle fibres (green) in an ESM cross section. Scale bar: 40 μm. (B) Immunostaining of dystrophin (magenta) in the sarcolemma of actinin^+^ muscle fibres (green) in an ESM cross section. Scale bar: 30 μm. (C) Transmission electron microscopy (TEM) images of sarcomere ultrastructure, T‐tubular triads and mitochondria along the muscle fibres in ESM. M, M line; Mt, mitochondria; TT, T‐tubule. Scale bar: 1 μm (left and middle panels) and 250 nm (right panel)

Enhanced functional maturation was associated with an advanced degree of structural maturation. Proteins of the DGC complex, such as β‐dystroglycan and dystrophin, were properly localized to the cell membrane (*Figure* 6A,B). Ultrastructural analysis by transmission electron microscopy showed advanced stages of myofibrillogenesis. Organized sarcomeres with distinct banding pattern including I bands, A bands, M lines and Z discs were observed, and mitochondria with dense matrix and developed cristae were found aligned with compact sarcomeres. In addition, membranous structures of the triad, composed of a central T‐tubule surrounded by two terminal cisternae from the sarcoplasmic reticulum, were identified (*Figure* 6C).

### Engineered skeletal muscles contain satellite‐like cells with regenerative capacity

**Figure 7:**
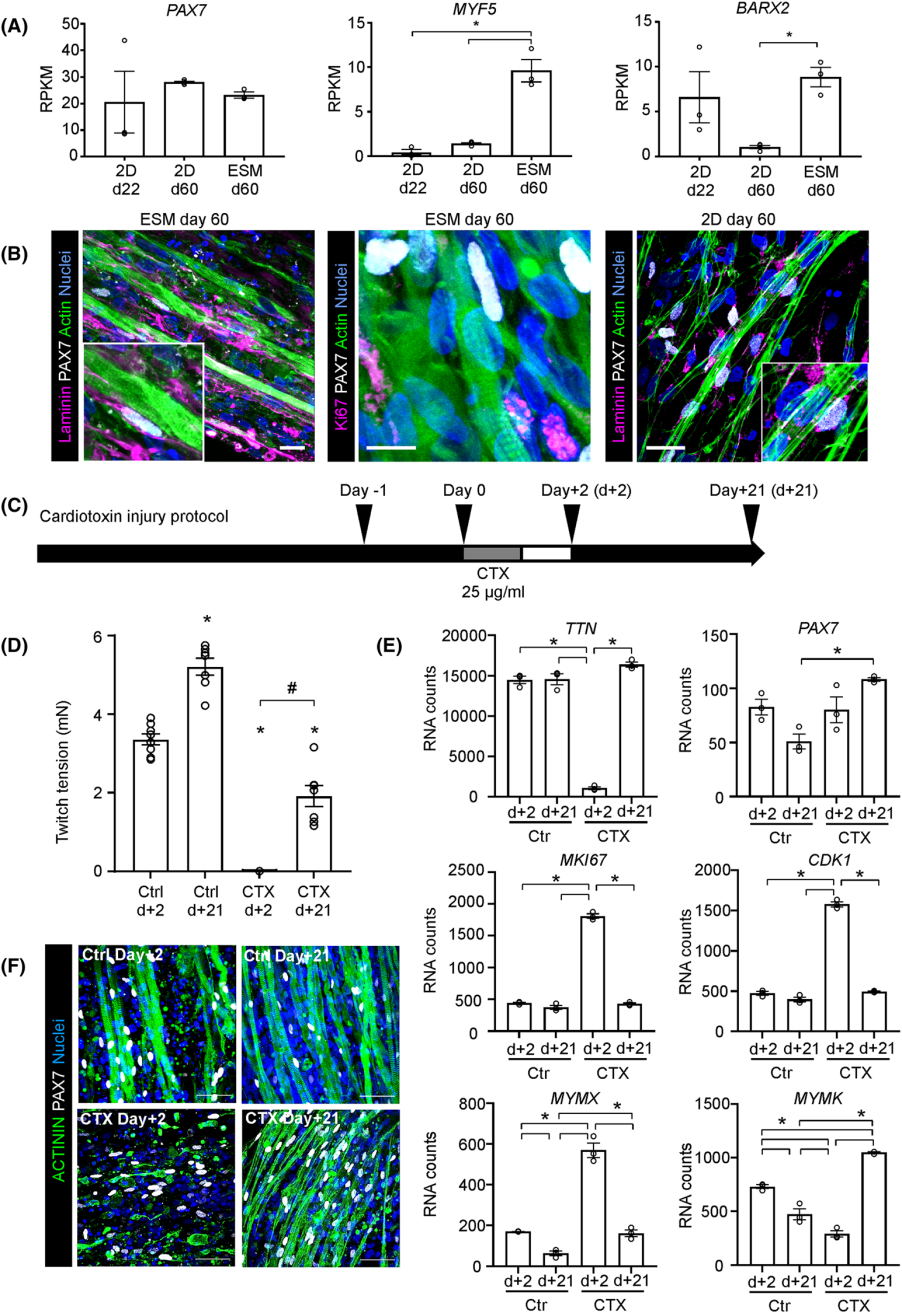
Regenerative capacity of human engineered skeletal muscle. (A) RNA transcript (reads per kilobase million [RPKM]) of indicated muscle stem cell markers in 2D monolayer cells at Day 22 and Day 60, plus Day 60 ESM; *n* = 3–4 per group, ^*^ *P* < 0.05 by one‐way analysis of variance (ANOVA) and Tukey's multiple comparison test. (B) Immunostaining of longitudinal sections of Day 60 ESM for laminin (magenta), Ki67 (magenta), actin (green), PAX7 (grey) and nuclei (blue). Scale bars: 10 μm. Immunostaining of laminin (magenta), PAX7 (grey), actin (green) and nuclei (blue) in 2D monolayer cultures at Day 60. Scale bar: 50 μm. (C) Experimental design of cardiotoxin (CTX) injury model. ESMs were incubated with 25 μg/mL CTX for 24 h. (D) Tetanic twitch tension at 100 Hz stimulation frequency of ESM at indicated time points after CTX (25 μg/mL) injury or control (Ctrl) condition; *n* = 7–8 per group, ^*^ *P* < 0.05 versus the respective Ctrl Day +2, by one‐way ANOVA and Tukey's multiple comparison test, ^#^ *P* < 0.05 CTX Day +2 versus CTX Day +21. (E) RNA transcript abundance for indicated genes at early (d+2) and late (d+21) time points after CTX (25 μg/mL) injury or control (Ctrl) conditions; *n* = 3, ^*^ *P* < 0.05 by one‐way ANOVA and Tukey's multiple comparison test. (F) Immunostaining of sarcomeric α‐actinin (green), PAX7 (grey) and nuclei (blue) in ESM at indicated time points. Scale bars: 50 μm

Even after prolonged in vitro culture, we found muscle stem cell transcripts in the differentiated skeletal muscle cultures predominantly in 3D in line with the single‐nucleus sequencing data (*Figure* 7A). Immunostaining confirmed the presence of Pax7^+^ cells, 63 ± 4% (*n* = 267 cells counted) of which were located adjacent to a muscle fibre in ESM. The localization underneath the laminin^+^ basal lamina is indicative of a satellite cell position. Of note, 75 ± 6% (*n* = 164 cells counted) of these PAX7^+^ cells were Ki67^−^, confirming a quiescent state. In identically aged 2D monolayer cultures, only 32 ± 5% (*n* = 105 cells counted) of PAX7^+^ cells were associated with muscle fibres (*Figure* 7B). These data suggest that muscle cells in ESM self‐organize into myofibres with adjacent satellite‐like cells.

To test if the identified satellite cell‐like niches in ESM are capable of muscle regeneration, we applied a well‐established cardiotoxin (CTX) injury model (*Figure* 7C, ^27^); 2 days after CTX injury, ESM did not generate measurable contractile forces, indicative of a complete loss of organized muscle fibres. After a regeneration period of 21 days, a partial, but robust recovery of contractile force (to 57 ± 8% of initial force, *n* = 7) was observed (*Figure* 7D). RNA expression data were in line with the functional data showing an almost complete loss of mature muscle transcript (*TTN*), whereas *PAX7* transcript was largely preserved 2 days after CTX injury (*Figure* 7E). Upregulation of Ki67 (*MKI67*) and *CDK1* indicated cell cycle activation 48 h post‐injury coinciding with Myomixer (*MYMX*) and followed by Myomaker (*MYMK*) expression to indicate active fusion of myoblast progeny. Importantly, recovery of contractile force was paralleled by re‐expression of *TTN* muscle transcript 21 days post‐injury. Immunostaining confirmed the almost complete loss of mature myofibres with sparing of PAX7^+^ satellite‐like cells 2 days after CTX injury. After 21 days of regeneration, substantial muscle was re‐built (*Figure* 7F). Interestingly, we also observed an activation of MCs in ESM by CTX injury; 2 days post‐injury, mesenchymal transcripts *EBF2* and *FAP* were increased (*Figure* S5A). This was associated with detection of Ki67^+^ in both PAX7^+^ and PAX7^−^ cell populations (*Figure* S5B). To test if the cell cycle activation of satellite‐like cells is required for ESM regeneration, we inhibited cell cycle activity by irradiation with 30 Gy. This completely abolished the regenerative response and formation of new muscle fibres (*Figure* S6). Note that irradiation of uninjured muscle did not impact contractile force. Those data demonstrate that functional muscle regeneration can be modelled in ESM.

### Myoediting rescues contractile dysfunction of Duchenne muscular dystrophy engineered skeletal muscle

**Figure 8:**
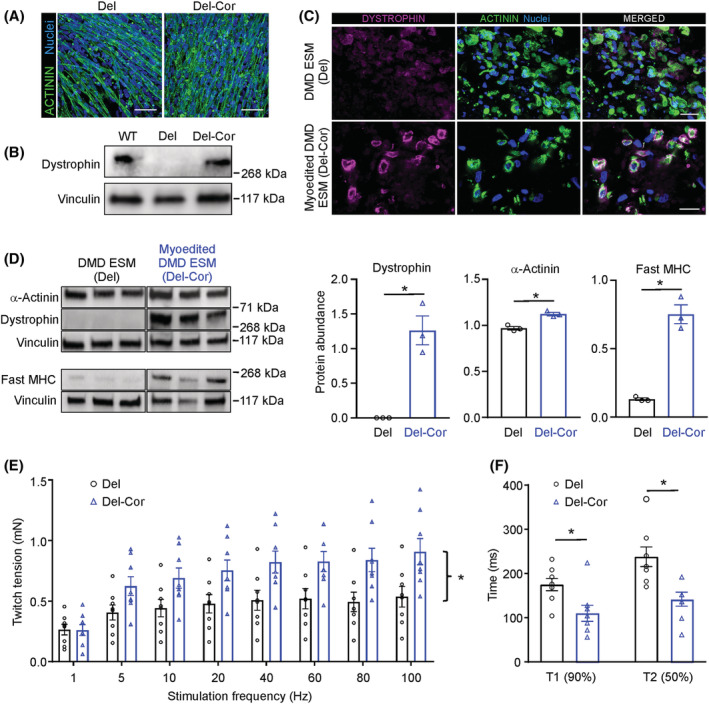
Modelling Duchenne muscular dystrophy in engineered skeletal muscle (ESM). (A) Immunostaining of sarcomeric α‐actinin (green) and nuclei of 2D monolayer skeletal muscle cells at Day 22 from Del and Del‐Cor lines. Scale bar: 50 μm. (B) Immunoblot for dystrophin in WT, Del and Del‐Cor skeletal myocytes. Vinculin serves as loading control. (C) Immunostaining of 5‐week‐old ESM cross sections for dystrophin (magenta), α‐actinin (green) and nuclei. Scale bar: 20 μm. (D) Immunoblot of ESM for α‐actinin, dystrophin and fast myosin heavy chain. Vinculin serves as loading control. Quantification of protein data, *n* = 3, ^*^ *P* < 0.05 by unpaired, two‐sided Student's *t* test. (E) Twitch tension in response to increasing stimulation frequencies of 5‐week‐old Del‐ESM (black bars) and Del‐Cor ESM (blue bars), *n* = 8 per group, ^*^ *P* < 0.05 by two‐way analysis of variance (ANOVA) and Tukey's multiple comparison test. (F) Quantification of contraction (T1) and relaxation (T2) times of single twitches of 5‐week‐old Del‐ESM (black bars) or Del‐Cor ESM (blue bars) ESM at 1 Hz. ^*^ *P* < 0.05 by unpaired, two‐sided Student's *t* test

To investigate if ESMs recapitulate contractile dysfunction of DMD, we generated ESMs from a patient‐derived iPSC line with a large exon 48–50 deletion (Del), leading to a premature stop codon in exon 51.^32^ This was compared to an isogenic control where the DMD reading frame was restored by CRISPR/Cas9‐mediated destruction (‘myoediting’) of the splice acceptor site of exon 51 (Del‐Cor, *Figure S6* ^32^). The absence of dystrophin protein did not have an impact on skeletal myocyte differentiation (*Figures* 8A and S2). We confirmed the absence of dystrophin in 2D differentiated skeletal myocytes and ESM from Del myocytes as well as its restoration after myoediting by immunostaining and western blot analyses (*Figure* 8B–D). Loss of dystrophin was associated with a reduction of fast MHC expression in DMD ESM in line with early reports on predominant affection of fast MHC‐expressing fibres in DMD muscles (*Figure* 8D, ^33^, ^34^). Total muscle content measured by α‐actinin was only slightly reduced (−13 ± 2%, *n* = 3, *P* < 0.05) in DMD ESM (*Figure* 8D). Importantly, loss of dystrophin and reduction of fast MHC resulted in reduced twitch tension (−35 ± 7% at 100 Hz tetanus, *n* = 8, *P* < 0.05) and prolonged contraction and relaxation times demonstrating that ESM culture unmasks early changes of DMD myopathy (*Figure* 8E,F).

## Discussion

We report a novel model for human skeletal muscle derivation in 2D and 3D organoid (SMO) cultures as well as for the engineering of skeletal muscle (ESM) with advanced structural and functional properties. Our data suggest that multicellularity (including neurons and supporting mesenchyme) and three‐dimensionality are key for in vitro skeletal muscle development with in vivo properties. The re‐engineering of a regeneration‐competent satellite‐like cell niche appears particularly interesting as it may not only offer a solution for disease modelling and drug screening but also for stable culture and amplification of muscle stem cells for regenerative applications (as demonstrated previously for the rat model^27^). The demonstration of enhanced maturation of the developed muscle model under T3 supplementation and uncovering of a DMD phenotype further demonstrate the applicability as a screening tool for maturation advancing factors and preclinical therapies in vitro.

In contrast to myogenic transcription factor overexpression models,^4^, ^5^, ^6^, ^7^, ^8^, ^9^, ^10^ we directed differentiation in 2D monolayer culture and 3D organoids using defined and developmentally inspired growth factors and small molecules. The strength of this approach is potential control over the developmental origin of resulting muscle. Following the induction of paraxial mesoderm, we demonstrated that maintaining FGF signalling in the presence of Notch inhibition (DAPT) induces a hypaxial dermomyotome pattern during in vitro somitogenesis between Days 8 and 13 with predominant expression of PAX3 and SIM1 but little EN1. EN1 expression increases at later time points in both 2D and 3D cultures consistent with its role in limb formation.^35^ The predominant development into limb muscle was further supported by increase in transcript and protein of migratory limb progenitors (*LBX1* and *MET*). Further dissecting and controlling developmental origin of muscle in vitro will be important to faithfully recapitulate disease states of particular muscle compartments (e.g., limb and diaphragm in DMD).

Although the SMO model, to our knowledge the first human SMO model with bona fide muscle function, allows for a simulation of embryonic muscle development, our data demonstrate that more classical tissue engineering models, such as applied for the generation of ESM, are more likely to achieve higher levels of organotypic maturation. ESM demonstrated higher myofibre diameter, clear anisotropic structure with membrane localized DGC, advanced ultrastructural properties (e.g., Z, I, A, H and M bands and T‐tubules) and ~2‐fold higher tetanic forces.

Despite the advanced organotypic properties, it is important to point out that the observed contractile parameters in ESM are not fully representative of adult skeletal muscle. For example, myofibres in ESM present with a smaller average muscle cell diameter (0.2‐ to 0.3‐fold), a still rather foetal myosin isoform expression pattern (high MYH3 to MYH2 ratio), ~10% of the maximal contractile force reported for adult muscle, predominantly central nuclei and a high number of progenitor cells (PAX7^+^). Strategies to enhance physiological hypertrophic growth are needed to further enhance skeletal muscle properties. Increased fast MYH2 and reduced MYH3 expression under exposure to T3 represent first proof of concept for the propensity of ESM to undergo further maturation if exposed to supportive stimuli. In this context, it is important to emphasize that the introduced fully serum‐free process will be advantageous for the testing of additional maturation factors.

We further demonstrate that the ESM model uncovers an apparent early developmental defect in DMD with reduction of fast myosin isoforms compared to the corrected isogenic line with dystrophin protein expression. This was associated with prolonged contraction and relaxation times in DMD muscle as well as reduced contractile force, supporting recent findings in functionalized monolayer cultures of DMD muscle cells.^22^ Importantly, in this model, we could clearly demonstrate the ‘therapeutic’ effect of CRISPR/Cas9‐based myoediting, demonstrating the general utility of ESM for the preclinical assessment of gene therapy strategies in myopathies.

Finally, the observation of regeneration in ESM after CTX‐induced damage in dependence of PAX7^+^ satellite‐like cell function was particularly notable, because it demonstrates that regeneration‐competent satellite‐like cells can be developed using the reported protocol. The recovery of twitch force is a comprehensive readout of regeneration as it integrates diverse aspects of the regenerative response, for example, activation and proliferation of muscle stem cells, fusion of myoblasts, maturation of regenerating fibres, but also ‘regenerative crosstalk’ to MCs, neurons or immune cells. The robust but incomplete recovery of twitch force after CTX injury needs further clarification but may point to the developmental status of ESM with a foetal stage of PAX7^+^ satellite cell development without the full regenerative potential of quiescent adult satellite cells that typically evolve after birth.^36^

The regeneration of engineered muscle by satellite‐like cell activation is fascinating and has only recently been observed for human muscle in vitro.^37^ Fleming et al. used primary muscle cells similar to earlier work in the rat^27^, ^38^ and a BaCl_2_ injury model, which may partially spare myotubes, leaving the possibility for PAX7^+^ cell‐independent regeneration.^39^ To avoid this limitation, we have carefully titrated CTX to destroy most if not all developed myofibres, while sparing only and most of the PAX7^+^ cells. The following sequelae of PAX7^+^ cell activation, proliferation and fusion were completely inhibited by irradiation, which supports a true regenerative pattern.

We conclude that the skeletal muscle differentiation protocols in monolayer culture and in a novel organoid format (SMO) as well as the demonstration of skeletal muscle tissue engineering (ESM) as a means to enhance maturation provide innovative platforms to study human skeletal muscle development, disease and regeneration in a simple and robust in vitro model.

## Conflicts of interest

The University of Göttingen has filed a patent on skeletal muscle generation listing M. Shahriyari, W‐H.Z. and M.T. as inventors (WO 2021/074126A1). W‐H.Z. is founder, shareholder and advisor of myriamed GmbH, MyriaMeat GmbH and Repairon GmbH. M.T. is founder of MyriaMeat GmbH and advisor of myriamed GmbH and Repairon GmbH.

## Supporting information


**Table S1.** Gene ontology (GO) enrichment and associated biological processes in coexpression clusters.
**Figure S1.** Identification of myogenic cell populations during directed differentiation of human PSC. Immunostaining of OCT4, PAX3, LBX1, PAX7, MYOD1, MYOGENIN, sarcomeric α‐actinin (in gray), and nuclei (blue) at indicated time points of skeletal muscle differentiation in 2D and 3D. Scale bar: 500 μm (2D), 50 μm (3D).
**Figure S2.** Efficiency of skeletal myocyte differentiation from human PSC. (A) Representative immunostaining of myogenic transcription factors: PAX7, MYOD1 and MYOGENIN (gray), actin (green) and nuclei (blue) in 22 days old skeletal muscle cultures from TC1133 (WT 1) line; Scale bars: 50 μm. Quantification of nuclei positive for PAX7, MYOD1 and MYOGENIN in 22 days old myogenic cultures from HES2 and from iPSC (WT 1) lines; *n* = 9–13 differentiations. (B) Flow cytometry of myogenic transcription factors PAX7, MYOD1, MYOGENIN in comparison to isotype control (IgG) in day 22 old skeletal myocyte cultures from one ESC line (HES2) and four iPSC lines (WT1, WT2, DMD Del, DMD Del‐Cor).
**Figure S3.** Comparison of ESM from PSC and primary skeletal myocytes. (A) Representative original twitch tension traces at 100 Hz tetanic contraction of ESM prepared from PSC‐derived (PSC ESM) or biopsy‐derived primary SkM (pSkM ESM). (B) 100 Hz tetanic twitch tension (TT) of ESM from HES2 (orange circle) and WT1, WT2 iPSC lines (black circles), and ESM from 6 different patient biopsies (pSkM ESM); *p < 0.05 by unpaired, two‐tailed Student's t‐test. (C) Transcript levels (RNA counts measured by nCounter) of indicated muscle genes in PSC ESM or pSkM ESM, *n* = 6, p < 0.05 by 2‐way ANOVA and Sidak's multiple comparison test. (D) Brightfield images of primary skeletal myoblast/myotube cultures after magnetic cell sorting for CD56 and PSC‐derived mesenchymal cells after magnetic cell sorting with anti‐Fibroblast beads. Scale bar: 100 μm. (E) 100 Hz tetanic twitch tension (TT) of ESM generated without or with addition of 20% PSC‐derived mesenchymal cells (MC), *n* = 4, *p < 0.05 by Student's t‐test.
**Figure S4.** Thyroid hormone elevates the tetanus threshold of ESM. (A) Fusion index calculated on representative traces of twitch tension generated by control (black) and +T3 (blue) ESM (9 wks old) at 5 Hz tetanus stimulation. The fusion index calculated as the percentage ratio of the maximal relaxation amplitude before the last contraction of the tetanus (C_min_) to the amplitude of this last contraction (C_max_). (B) The fusion index‐frequency curve of control (black line) and +T3 (blue line) ESM. *p < 0.05 by 2 way‐ANOVA and Tukey's multiple comparison test. (C) Stimulation frequency at 50% tetanus fusion of control (black bar) and +T3 (blue bar) ESM; *n* = 8/group, *p < 0.05 by Student's t‐test.
**Figure S5.** Activation of mesenchymal cells by CTX injury. (A) RNA transcript abundance for indicated non‐myocyte genes at early (d + 2) and late (day+21) time points after CTX (25 μg/ml) injury or control (Ctrl) conditions; *n* = 3, *p < 0.05 by 1‐way ANOVA and Tukey's multiple comparison test. (B) Immunostaining of injured ESM for Ki67 (magenta), actin (green), PAX7 (gray), and nuclei (blue). Arrows label Ki67^+^ PAX7^−^ cells, stars label Ki67^+^ PAX7^+^ cells; Scale bar: 20 μm.
**Figure S6.** Irradiation blocks regenerative capacity of human engineered skeletal muscle. (A) Experimental scheme of irradiation protocol. One day before cardiotoxin (CTX) injury ESM were irradiated with 30 Gy. ESM were then incubated with 25 μg/ml CTX for 24 hrs. (B) Tetanic twitch tension at 100 Hz stimulation frequency of ESM with irradiation at indicated time points after CTX (25 μg/ml) injury or control (Ctrl) condition; *n* = 3–4/group, *p < 0.05 vs. Ctrl day+2 by 1‐way ANOVA and Tukey's multiple comparison test. (C) Immunostaining of sarcomeric α‐actinin (green) and nuclei (blue) in non‐irradiated ESM (top panel) and irradiated ESM (bottom panel) 21 days after CTX injury. Scale bars: 50 μm.
**Figure S7.** Modeling Duchenne muscular dystrophy in ESM. Scheme of experimental setup.
**Table S3.** List of antibodies

**Table S2.** Enrichment of snSeq clusters

**Table S4.** Cell group genes

**Video S1:** Spontaneous contractions 2D skeletal myocyte cultures (day 30)

**Video S2:** Spontaneous contractions of SMO matured for 3 weeks on metal holders

**Video S3:** Spontaneous contractions of ESM matured for 2 weeks on metal holders
