# Supplementary material for: Engineered skeletal muscle recapitulates human muscle development, regeneration and dystrophy
Source: J Cachexia Sarcopenia Muscle. 2022 Oct 18;13(6):3106–21. doi: 10.1002/jcsm.13094 (PMC9745484; doi:10.1002/jcsm.13094)
Supplement: Supplementary file 1 — Table S1. Gene ontology (GO) enrichment and associated biological processes in coexpression clusters. Figure S1. Identification of myogenic cell populations during directed differentiation of human PSC. Immunostaining of OCT4, PAX3, LBX1, PAX7, MYOD1, MYOGENIN, sarcomeric α‐actinin (in gray), and nuclei (blue) at indicated time points of skeletal muscle differentiation in 2D and 3D. Scale bar: 500 μm (2D), 50 μm (3D). Figure S2. Efficiency of skeletal myocyte differentiation from human PSC. (A) Representative immunostaining of myogenic transcription factors: PAX7, MYOD1 and MYOGENIN (gray), actin (green) and nuclei (blue) in 22 days old skeletal muscle cultures from TC1133 (WT 1) line; Scale bars: 50 μm. Quantification of nuclei positive for PAX7, MYOD1 and MYOGENIN in 22 days old myogenic cultures from HES2 and from iPSC (WT 1) lines; n = 9–13 differentiations. (B) Flow cytometry of myogenic transcription factors PAX7, MYOD1, MYOGENIN in comparison to isotype control (IgG) in day 22 old skeletal myocyte cultures from one ESC line (HES2) and four iPSC lines (WT1, WT2, DMD Del, DMD Del‐Cor). Figure S3. Comparison of ESM from PSC and primary skeletal myocytes. (A) Representative original twitch tension traces at 100 Hz tetanic contraction of ESM prepared from PSC‐derived (PSC ESM) or biopsy‐derived primary SkM (pSkM ESM). (B) 100 Hz tetanic twitch tension (TT) of ESM from HES2 (orange circle) and WT1, WT2 iPSC lines (black circles), and ESM from 6 different patient biopsies (pSkM ESM); *p < 0.05 by unpaired, two‐tailed Student's t‐test. (C) Transcript levels (RNA counts measured by nCounter) of indicated muscle genes in PSC ESM or pSkM ESM, n = 6, p < 0.05 by 2‐way ANOVA and Sidak's multiple comparison test. (D) Brightfield images of primary skeletal myoblast/myotube cultures after magnetic cell sorting for CD56 and PSC‐derived mesenchymal cells after magnetic cell sorting with anti‐Fibroblast beads. Scale bar: 100 μm. (E) 100 Hz tetanic twitch tension (TT) of ESM gen [file JCSM-13-3106-s003.docx]

**SUPPLEMENTARY MATERIAL**

**Engineered skeletal muscle recapitulates human muscle development, regeneration, and dystrophy**

Mina Shahriyari^1,2^, Md Rezaul Islam^3^, M Sadman Sakib^3^, Malte Rinn^1,2^, Anastasia Rika^1,2^, Dennis Krüger^3^, Lalit Kaurani^3^, Verena Gisa^3^, Mandy Winterhoff^1,2^, Harithaa Anandakumar^1,2^, Orr Shomroni^4^, Matthias Schmidt^5^, Gabriela Salinas^4^, Andreas Unger^6^, Wolfgang A. Linke^6^, Jana Zschüntzsch^5^, Jens Schmidt^5,7^, Rhonda Bassel-Duby^8,9,10^, Eric N. Olson^8,9,10^, André Fischer^3,11^, Wolfram-Hubertus Zimmermann^1,2,11,12*^, Malte Tiburcy^1,2*^

^1^ Institute of Pharmacology and Toxicology, University Medical Center Göttingen, Göttingen, Germany.

^2^ DZHK (German Center for Cardiovascular Research), partner site Göttingen, Germany

^3^ Department for Epigenetics and Systems Medicine in Neurodegenerative Diseases, German Center for Neurodegenerative Diseases (DZNE) Göttingen, Göttingen, Germany

^4^ NGS Integrative Genomics Core Unit, Institute of Human Genetics, University Medical Center Göttingen, Göttingen, Germany

^5^ Department of Neurology, University Medical Center Göttingen, Göttingen, Germany

^6^ Institute of Physiology II, University of Münster, D-48149 Münster, Germany

^7^ Department of Neurology and Pain Treatment, University Hospital of the Medical School Brandenburg, Immanuel Klinik Rüdersdorf, Rüdersdorf bei Berlin, Germany

^8^ Department of Molecular Biology, University of Texas Southwestern Medical Center, Dallas, TX 75390, USA.

^9^ Senator Paul D. Wellstone Muscular Dystrophy Cooperative Research Center, University of Texas Southwestern Medical Center, Dallas, TX 75390, USA.

^10^ Hamon Center for Regenerative Science and Medicine, University of Texas Southwestern Medical Center, Dallas, TX 75390, USA

^11^ Cluster of Excellence "Multiscale Bioimaging: from Molecular Machines to Networks of Excitable Cells" (MBExC), University of Göttingen, Germany

^12^ Fraunhofer Institute for Translational Medicine and Pharmacology (ITMP), Göttingen, Germany

**SUPPLEMENTARY METHODS**

**Human pluripotent stem cell culture**

The following pluripotent stem cell (PSC) lines were used in the study: Wild-type iPSC TC1133 (WT1;[1]), Wild-type iPSC iPSC-line 2 (WT2) from iPieran, previously reported in Kamdar et al. and Darabi et al. [2, 3], DMD iPSC Del48-50 (DMD Del; purchased from RIKEN BioResource Center, HPS0164), corrected DMD iPSC Del48-50 (DMD Del-Cor [4], and HES2 (WiCell). The use of HES2 line was approved by the Robert-Koch-Institute (3.04.02/0160). Informed consent and ethical approval by the University Medical Center Göttingen was obtained for use of human iPSC lines (10/9/15). All lines were routinely tested for pluripotency and confirmed to be free of mycoplasma (Lonza Mycoalert™ kit).

**Primary human myoblast culture**

Muscle samples (Erector spinae muscle) were taken from patients during spine surgery after obtaining informed consent and with ethical approval by the University Medical Center Göttingen (5/2/16). Human muscle cell progenitors (satellite cells) were isolated according to the following protocol [5]. In short, the muscle piece was minced and washed in phosphate buffered saline and trypsinized. The fragments were seeded to a 25-cm^2^ flask in skeletal muscle growth medium with supplement mix (PromoCell) and 1% Pen/Strep. After 21 days, myoblasts were labeled with anti-CD56/NCAM (mouse clone Eric-1; Thermo Fisher Scientific), followed by magnetic bead–labeled secondary antibodies and subsequently separated by magnets (Dynal/Invitrogen). For further expansion cells were seeded to T175 cell culture flasks in skeletal growth medium, an expanded for an average 70±16 days (n=5).

**Magnetic cell sorting of iPSC skeletal muscle mesenchymal cells**

To separate the mesenchymal cell fraction from myogenic and neural cells, differentiated skeletal muscle cultures between day 22 and 29 were enzymatically dispersed with TrypLE Express (Thermo Fisher Scientific) for 4 min at 37°C. The cells were gently resuspended in N2-HK medium containing 5 µmol/L Y27632. After straining through a 70 µm cell strainer (Sarstedt), 10^7^ cells were pelleted at 300 x g for 10 min. The cell pellet was resuspended in 80 µL in BD FACS Pre-sort buffer (BD Biosciences) to which 20 µL of Anti-Fibroblast MicroBeads (Miltenyi Biotec) were added. The cells were incubated at room temperature for 30 min followed by a wash with 2 mL of Pre-sort buffer. The pellet was resuspended in 500 µL of pre-sort buffer and separated on 2 consecutive LS columns (Miltenyi Biotec) that were placed in a QuadroMACS separator (Miltenyi Biotec) according to the manufacturer’s instruction. The positive cell population was then plated and expanded in Endothelial Cell Growth Medium MV2 (Promocell).

### **Preparation of casting molds and static stretchers for ESM and SMO generation**

For the generation of the 3D muscle models, poly-dimethylsiloxane (PDMS; SYLGARD TM 184 Silicone Elastomer Kit, Dow Corning) circular molds with inner/outer diameter 4/6 mm and 2.5 mm height were fabricated and allowed to cure overnight at 55°C. Static stretch devices were made from a Teflon® base and stainless-steel holders. The detailed protocols for the preparation of the casting molds and static stretchers have been described previously [6, 7].

**Generation of human skeletal muscle organoids (SMOs)**

To make skeletal muscle organoids (SMOs) from PSCs a final 250 µl/SMO volume mixture of (i) 36 µl of 6.5 mg/ml (final amount of 0.23 mg) acid soluble collagen type 1 (LLC Collagen Solutions), (ii) 36 µl of concentrated 2x DMEM (Thermo Fisher Scientific) serum-free medium (0.27 g DMEM powder DMEM, powder, low glucose, pyruvate in 10 ml ddH2O), (iii) 6.75 µl of NaOH 0.1 N (Carl Roth), (iv) 10% v/v Matrigel™ (BD) and v) 0.8 x 10^6^ PSC resuspended in 157.5 µl of StemMACS iPS-Brew XF (Miltenyi Biotec) medium with 5 µmol/L Y27632 (Stemgent), 10 ng/ml FGF-2 (Peprotech) and 10% knockout serum replacement (ThermoFisher Scientific) was cast into circular polydimethylsiloxane (PDMS) molds. After 1 h of hydrogel polymerization at 37°C, StemMACS iPS-Brew XF (Miltenyi Biotec) medium with 5 µmol/L Y27632 (Stemgent), 10 ng/ml FGF-2 (Peprotech) and 10% Knockout serum replacement (ThermoFisher Scientific) was added for 24 hrs. After tissue compaction skeletal muscle differentiation was induced following the protocol established in 2D. On day 22 of differentiation, SMOs were loaded on static stretchers at 120% of slack length and cultured in maturation medium for 4 additional weeks.

**Generation of human engineered skeletal muscle (ESM)**

A final 250 µl/ESM volume mixture of (i) 36 µl of 6.5 mg/ml (final amount of 0.23 mg) acid soluble collagen type 1 (Collagen Solutions), (ii) 36 µl of concentrated 2x DMEM (Thermo Fisher Scientific) serum-free medium (0.27 g DMEM, powder, low glucose, pyruvate in 10 ml ddH2O), (iii) 6.75 µl of NaOH 0.1 N (Carl Roth), (iv) 10% v/v Matrigel™ (BD) and (v) 1.25 x 10^6^ of day 22 human PSC-derived skeletal myocytes which were resuspended in 157.5 µl of expansion medium with 5 µmol/L Y27632 (Stemgent), was cast into circular PDMS molds. After 1h of polymerization at 37°C, ESMs were cultured in expansion medium with 5 µmol/L Y27632 (Stemgent) for 24h and then expansion medium for another 6 days to compact into mechanically stable tissue. After transfer of ESMs onto static stretchers they were cultured in maturation medium under mechanical load up to 9 weeks. For generation of engineered muscle from primary myoblasts, cells were cast in a final 250 µl/ESM volume mixture of (i) 36 µl of 6.5 mg/ml (final amount of 0.23 mg) acid soluble collagen type 1 (Collagen Solutions), (ii) 36 µl of concentrated 2x DMEM (Thermo Fisher Scientific) serum-free medium (0.27 g DMEM, powder, low glucose, pyruvate in 10 ml ddH2O), (iii) 6.75 µl of NaOH 0.1 N (Carl Roth), (iv) 10% v/v Matrigel™ (BD) and (v) 1.25 x 10^6^ of primary skeletal myoblasts and cultured on static stretchers in maturation medium after compaction.

**Isometric force measurements**

Contractile function of ESM/SMO was measured under isometric conditions in a thermostatted organ bath (Föhr Medical Instruments) filled with gassed (5% CO_2_/95% O_2_) Tyrode’s solution (containing: 120 NaCl, 1 MgCl2, 0.2 CaCl2, 5.4 KCl, 22.6 NaHCO3, 4.2 NaH2PO4, 5.6 glucose, and 0.56 ascorbate; all in mmol/L) at 37°C as described previously [8, 9]. The calcium concentration was set to 1.8 mM. To normalize for the force-length relationship, muscles were extended to Lmax (length of maximal twitch tension) under electrical stimulation with 1 Hz with 4 ms square pulses of 200 mA. At Lmax, twitch tension was assessed at varying frequencies (4-second long stimulation at 1, 5, 10, 20, 40, 60, 80 and 100 Hz). Contraction data was recorded with BMON software and analyzed using AMON software (Ingenieurbüro Jäckel). Single twitch kinetics were evaluated by time to peak contraction (T1: time from 10% to peak contraction) and time to 50% relaxation (T2: time from peak contraction to 50% relaxation).

**Cardiotoxin injury model**

To induce muscle injury ESM was incubated with 25 µg/ml of of *Naja pallida* cardiotoxin (CTX; Latoxan) for 24 h in maturation medium [10]. Subsequently the injured tissue was rinsed and cultured in expansion medium for 1 week followed by maturation medium for another 2 weeks of regeneration. Medium was refreshed every second day. To irradiate the ESM prior to CTX injury the culture plate was placed in an STS Biobeam 8000 gamma irradiator and exposed to a single dose of 30 Gy irradiation over 10 minutes [10].

**Morphological analyses**

2D cell cultures were fixed in 4% formalin (Carl Roth) at 20-22°C for 15 min. ESM/SMO were fixed in 4% formalin at 4°C overnight. After 2 washes with PBS, ESM/SMO were embedded in 2% agarose (peqGOLD) in 1X Tris Acetate-EDTA (TAE) buffer. Using the Leica Vibrotome (LEICAVT1000S), sections were cut at 400 µm and kept in cold PBS. Prior to staining, 2D cell cultures as well as ESM sections were washed with PBS. For blocking and permeabilization, samples were incubated in blocking buffer (PBS with 5% fetal bovine serum, 1% bovine serum albumin (BSA), and 0.5% Triton-X). All the primary and secondary antibody staining was performed in blocking buffer (please see **Supplementary Table 3** for list of antibodies). After 3x PBS washes for 5 minutes, the appropriate Alexa Fluor-coupled secondary antibodies (1:1000, Thermo Fisher Scientific) were applied for 2h at 20-24°C. In parallel with secondary antibodies, Alexa Fluor 633-conjugated phalloidin (1:100, Thermo Fisher Scientific), Alexa Fluor 594-conjugated α-Bungarotoxin (Thermo Fisher Scientific), and Hoechst-33342 (1:1000, Molecular Probes) were added to stain f-actin, nicotinic acetylcholine receptors, and nuclei, respectively. Following 3 washes with PBS, samples were mounted in Fluoromount-G (Southern Biotech). All the images were acquired by using a Zeiss LSM 710/NLO confocal microscope. Quantitative analyses of immunostainings were done with ImageJ cell counter tool.

**Transmission Electron Microscopy**

Ultrastructural analysis was performed on ESM samples fixed in 4% formalin (Carl Roth), 15% saturated picric acid in 0.1 M PBS, pH 7.4, at 4°C overnight. ESMs were rinsed twice with PBS and treated with 0.5% OsO_4_ for 45 min following several washing steps in 100 mM phosphate buffer. Samples were counterstained with uranyl acetate, dehydrated via ethanol series, and embedded in Durcupan ACM epoxy resin (Sigma-Aldrich). Ultrathin sections were prepared from resin blocks using a Leica Ultracut S ultramicrotome (Mannheim, Germany) and adsorbed to glow-discharged formvar-carbon–coated copper single-slot grids. Electron micrographs were recorded using a Zeiss LEO 910 electron microscope; images were taken with a TRS Sharpeye CCD camera (Troendle, Moorenweis, Germany).

**Flow cytometry**

Cells were fixed in 4% formalin (Carl Roth) at 20-22°C for 15 min. Following 2X washes with PBS, fixed samples were kept on ice for the staining process. Cells were incubated in blocking buffer (PBS with 5% fetal bovine serum, 1% bovine serum albumin (BSA), and 0.5% Triton-X) for 10 min followed by incubatin with primary antibodies (please see **Supplementary Table 3**) or isotype control for 45 min. After 2 washed with PBS appropriate secondary antibodies (1:1000, Thermo Fisher Scientific) were applied for 30 min. Samples were stained with Hoechst-33342 for nuclear DNA counting and exclusion of cell doublets. Cells were run on a LSRII cytometer and at least 10,000 events per sample were analyzed using Diva software (BD Biosciences).

**Western blotting**

For protein isolation snap frozen ESM or monolayer cells were homogenized in 150 µl of ice-cold protein lysate buffer (2.38 g HEPES, 10.20 g NaCl, 100 ml Glycerol, 102 mg MgCl2, 93 mg EDTA, 19 mg EGTA, 5 ml NP-40 in a total volume of 500 ml ddH2O) containing phosphatase and protease inhibitor cocktail (Roche) then centrifuged for 30 min at 12000 rpm at 4°C. 30 µg of protein sample was loaded onto a 4 to 15% gradient sodium dodecyl sulfate (SDS)-polyacrylamid gel (Bio-Rad), followed by protein transfer to a polyvinylidene fluoride (PVDF) membrane. To visualize the total protein, the PVDF membrane was stained with Ponceau Red. Primary antibody (4 h at 20-22°C) and secondary antibody (1 h in at 20-22°C) staining was performed in blocking solution containing 5% milk in 1x Tris-buffered saline (TBS) and 0.1% Tween 20. Please see **Supplementary Table 3** for antibody details. Protein loading was controlled by vinculin (VCL) protein content. The blots were developed with Femto LUCENT^TM^Luminol Reagent (Gbiosciences) and the protein bands were imaged using the Bio-Rad ChemDoc^TM^MP system. Protein quantification was performed using Image lab 6.1 (Bio-Rad).

**RNA expression analysis**

RNA was purified using Trizol (Thermo Fisher Scientific) according to the manufacturer’s instructions and quantified using Nanodrop. To analyze skeletal muscle specific transcripts an nCounter Elements TagSet panel from was designed by NanoString Technologies. Fifty nanograms of RNA per sample were hybridized to target-specific capture and reporter probes at 67 °C overnight (20 h) according to the manufacturer’s instructions. Samples were then loaded into the NanoString cartridge and nCounter Gene Expression Assay started immediately. Raw reads were analyzed with nSolverTM Data Analysis Software. Background subtraction was performed using geometric means of negative controls. RNA counts were normalized to 4 housekeeping genes (TBP, HPRT1, POL2RA, GAPDH).

**RNA sequencing**

Prior to sequencing RNA quality was ensured with the Fragment Analyzer from Advanced Analytical by using the standard sensitivity RNA Analysis Kit (DNF-471). RNA-seq libraries were prepared using a modified strand-specific, massively-parallel cDNA sequencing (RNA-Seq) protocol from Illumina, the TruSeq Stranded Total RNA. Libraries were sequenced on a HiSeq 4000 platform (Illumina) generating 50 bp single-end reads (30-40 Mio reads/sample). Sequence images were transformed with Illumina software BaseCaller to BCL files, which was demultiplexed to fastq files with bcl2fastq v2.17.1.14. The quality check was done using FastQC (version 0.11.5). Sequence reads were aligned to the human genome reference assembly (UCSC version hg38) using Star (version 2.5.2a) [11]. For each gene, the number of mapped reads was counted for human genes in ENSEMBL annotation hg38 version 89 using featureCounts (version 1.4.5) [12]. Raw counts were normalized and transformed to log2 counts per million (CPM) values. Reads Per Kilobase per Million mapped reads (RPKM) were calculated based on Ensembl transcript length using biomaRT (v2.24). All RNA sequencing data has been deposited in a public data base (GSE178270).

**Weighted co-expression analysis**

Weighted gene co-expression network analysis was performed using (WGCNA) package (version 1.61) in R. Briefly, normalized counts were transformed into log (base 2) counts and were used to calculate pairwise bi-weighted mid-correlations between genes. Next, based on approximate scale-free topology a soft threshold power of 14 was chosen and was used to calculate pair-wise topological overlap between genes to construct a signed gene co-expression network. Modules of co-expressed genes was later identified based on following criteria: minimum module size of 100, method = “hybrid”, deepSplit =0, pamRespectsDendro =T, pamStage = T. Modules with correlation higher than 0.85 were merged together. Different modules were summarized as modular eigengenes (MEs), those were then used to compare expression of the given module across differentiation time points. The module specific genes were further filtered based on a module membership correlation coefficient cutoff of 0.60. Gene ontology of the modules were analyzed using clusterProfiler (v3.0.4) and after multiple adjustments only statistically significant gene ontology terms (FDR <0.05) were retrieved. For pathway analysis, Reactome (https://reactome.org/) database was used.

**Published dataset analysis**

Raw data set from a previous study [13] was retrieved from NCBI GEO (accession: GSE90876) and processed as follows: sequencing reads were mapped to human genome hg38 using STAR aligner (v2.5.2b). After mapping, raw count files were generated using featureCounts of subread package (v1.5.1). For differential expression analysis, all samples were processed together and genes with less than 5 reads in 50% of the samples were filtered out prior to the analysis. Differential expression analysis was performed using DESeq2 package (version 1.28.1) in R. Genes with FDR < 0.05 were considered as differentially expressed. To test above chance overlap between previously identified module and differentially expressed genes, Fisher´s exact test was performed.

## **Single cell transcriptomics by single nuclei RNA sequencing**

Single nuclei were isolated from flash frozen cells or tissue. The sample was homogenized using a plastic pestle in a 1.5 ml Eppendorf tube containing 500 µl EZ prep lysis buffer (Sigma, NUC101-1KT) with 30 strokes. The homogenate was transferred into 2 ml microfuge tubes, lysis buffer was added up to 2 ml and incubated on ice for 7 minutes. After centrifuging for 5 minutes at 500xg supernatant was removed and the nuclear pellet was resuspended into 2 ml lysis buffer and incubated again on ice (7 minutes). After centrifuging for 5 minutes at 500xg, the supernatant was removed and the nuclei pellet was resuspended into 500 µl nuclei storage buffer (NSB: 1x PBS; Invitrogen, 0.5% RNase free BSA;Serva, 1:200 RNaseIN plus inhibitor; Promega, 1x EDTA-free protease inhibitor; Roche) and filtered through 40 μm filter (BD falcon) with additional 100 μL NSB to collect residual nuclei from the filter. Isolated nuclei were stained with a nuclear stain (7AAD) and FACS sorted (BD FACSaria III) to ensure a homogenous and viable nucleus preparation. Sorted nuclei were counted in a Countess FL II automated cell counter (ThermoFischer AMQAF1000, DAPI light cube: ThermoFischer: AMEP4650) with DAPI staining and nuclei concentration was adjusted to 1000 nuclei/μL. The nuclei were further diluted to capture and barcode 4000 nuclei according to Chromium single cell 3ʹ reagent kit v3 (10X Genomics). Single nuclei barcoding, GEM formation, reverse transcription, cDNA synthesis and library preparation were performed according to 10X Genomics guidelines. cDNA libraries were pooled and sequenced 4 times in Illumina NextSeq 550 in order to achieve the target reads / nuclei. Each sequencing run was acquiring 150bp paired-end reads (Illumina NextSeq 550 High Output Kit v2.5). Demultiplexing, read mapping (to pre-mRNA reference genome) and gene counts per nuclei were computed with cellranger (v4.0) software. The nuclei barcoding and sequencing pipeline typically allows to obtain 50.000-100.000 reads/nucleus resulting in detection of 200-10.000 genes/nucleus (median: ~2000 genes/nucleus) for further downstream analysis.

### **Bioinformatic analysis of single-nucleus RNA-sequencing**

Gene counts were obtained by aligning reads to the hg38 genome (NCBI:GCA 000001405.22) (GRCh38.p7) using CellRanger software (v.6.1.2) (10XGenomics). The CellRanger count pipeline was used to generate a gene-count matrix by mapping reads with the '--include-intron' option to account for unspliced nuclear transcripts. The SCANPY package was used for pre-filtering, normalization and clustering [14]. Initially, cells that reflected low-quality cells (based on read number and expression of house-keeping genes [15]) were excluded. Next, counts were scaled by the total library size multiplied by 10.000, and transformed to log space. Highly variable genes were identified based on dispersion and mean, the technical influence of the total number of counts was regressed out, and the values were rescaled. Principal component analysis (PCA) was performed on the variable genes, and UMAP was run on the top 50 principal components (PCs) [16]. The top 50 PCs were used to build a k-nearest-neighbours cell–cell graph with k= 100 neighbors. Subsequently, spectral decomposition over the graph was performed with 50 components, and the Leiden graph-clustering algorithm was applied to identify cell clusters. We confirmed that the number of PCs captures almost all the variance of the data. To assign cell-type identity of the identified clusters, the enriched genes of each cluster were subjected to GO term analysis using ShinyGO. Cluster with overlapping GO terms were combined to cell groups. Cell groups were then visualized by differentially expressed gene panels (log2FC > 2 and an adjusted p value of <0.05) of the contributing clusters (**Supplementary Table 4**).

**SUPPLEMENTARY TABLES**

**Supplementary Table 1.** Gene ontology (GO) enrichment and associated biological processes in coexpression clusters.

| **Cluster identifier** | **Enriched GO terms** | **Associated biological process** |
| --- | --- | --- |
| #1 | Muscle organ development | Migrating limb progenitors |
| #2 | No enrichment | None |
| #3 | Muscle tissue development | Somitogenesis |
| #4 | Cilium organization | Cilium organization |
| #5 | Regulation of neuron projection development; Muscle contraction | Muscle maturation |
| #6 | Hexose metabolic process | Muscle progenitor expansion |
| #7 | Ribonucleoprotein complex biogenesis | Translation |
| #8 | Response to endoplasmic reticulum stress | Sarcomerogenesis |
| #9 | Autophagy; Response to unfolded protein | Unfolded protein response |
| #10 | Mitochondrial gene expression | Primitive streak development |
| #11 | Lipid storage | Lipid storage |
| #12 | No enrichment | None |
| #13 | Oxidative phosphorylation | Cellular respiration |
| #14 | ncRNA metabolic process | Translation |
| #15 | Cilium organization | Presomitic progenitor development |
| #16 | No enrichment | None |
| #17 | Histone modification | Histone modification |
| #18 | Telomere maintenance via telomerase | Pluripotency |
| #19 | Regulation of cell cycle phase transition | Presomitic progenitor development |
| #20 | No enrichment | None |
| #21 | DNA conformation change | DNA organization |
| #22 | DNA replication; Nuclear division | Presomitic progenitor patterning |

**Supplementary Table 2. Enrichment of snSeq clusters**

**Supplementary Table 3. List of antibodies**

| **Antibodies** | | |
| --- | --- | --- |
| α-actinin (Sarcomeric) | Sigma-Aldrich | A7811 |
| Beta-Dystroglycan | Leica Biosystem | NCL-b-DG |
| Dystrophin Monoclonal | Sigma-Aldrich | MANDYS8 |
| Goat anti-mouse Alexa Fluor 488 | Thermo Fisher Scientific | A32723 |
| Goat anti-mouse Alexa Flour 546 | Thermo Fisher Scientific | A-11030 |
| Goat anti-rabbit Alexa Fluor 546 | Thermo Fisher Scientific | A-11035 |
| HRP-conjugated goat anti-mouse-IgG | Dako | P0161 |
| Ki67 | Abcam | Ab15580 |
| Laminin | Sigma-Aldrich | L 9393 |
| Lbx1 | Novusbio | NBP2-55646 |
| MyoD | Dako | MA1-41017 |
| Myogenin | DSHB | F5D |
| Myosin heavy chain 2 (fast type) | DSHB | A4.74 |
| Myosin heavy chain 3 (embryonic) | DSHB | F1.652 |
| Myosin heavy chain 7 (slow type) | DSHB | A4.951 |
| NCAM/CD56 | Thermo Fisher Scientific | **MA1-34183** |
| Oct4 | Abcam | Ab19857 |
| Pax3 | DSHB | Pax3-c |
| Pax7 | DSHB | Pax7-c |
| Vinculin (VCL) | Sigma-Aldrich | V9264 |

**Supplementary Table 4. Cell group genes**

**SUPPLEMENTARY FIGURES**


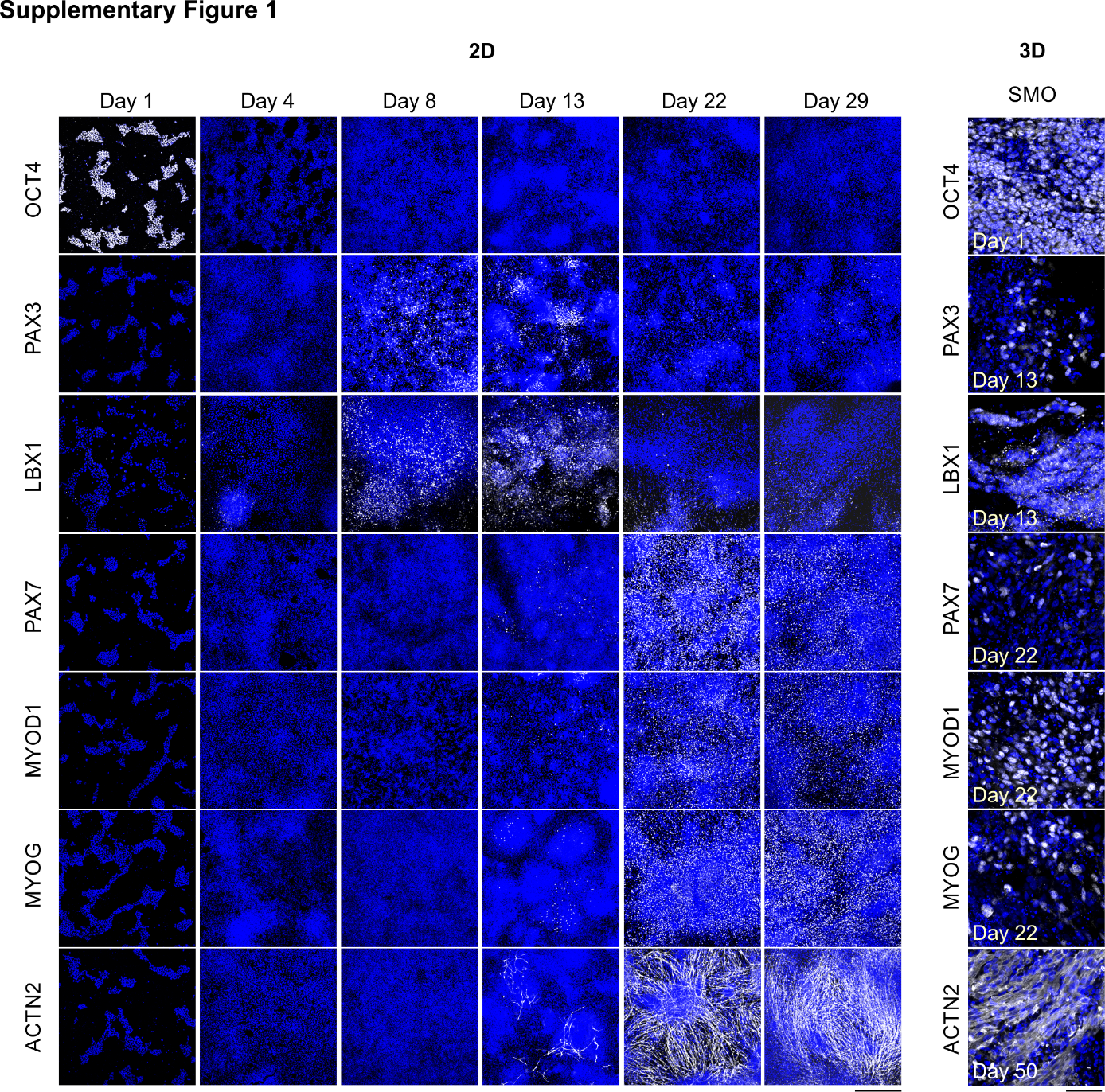


**Supplementary Figure 1. Identification of myogenic cell populations during directed differentiation of human PSC.** Immunostaining of OCT4, PAX3, LBX1, PAX7, MYOD1, MYOGENIN, sarcomeric α-actinin (ACTN2, in gray), and nuclei (blue) at indicated time points of skeletal muscle differentiation in 2D and 3D. Scale bar: 500 µm (2D), 50 µm (3D).


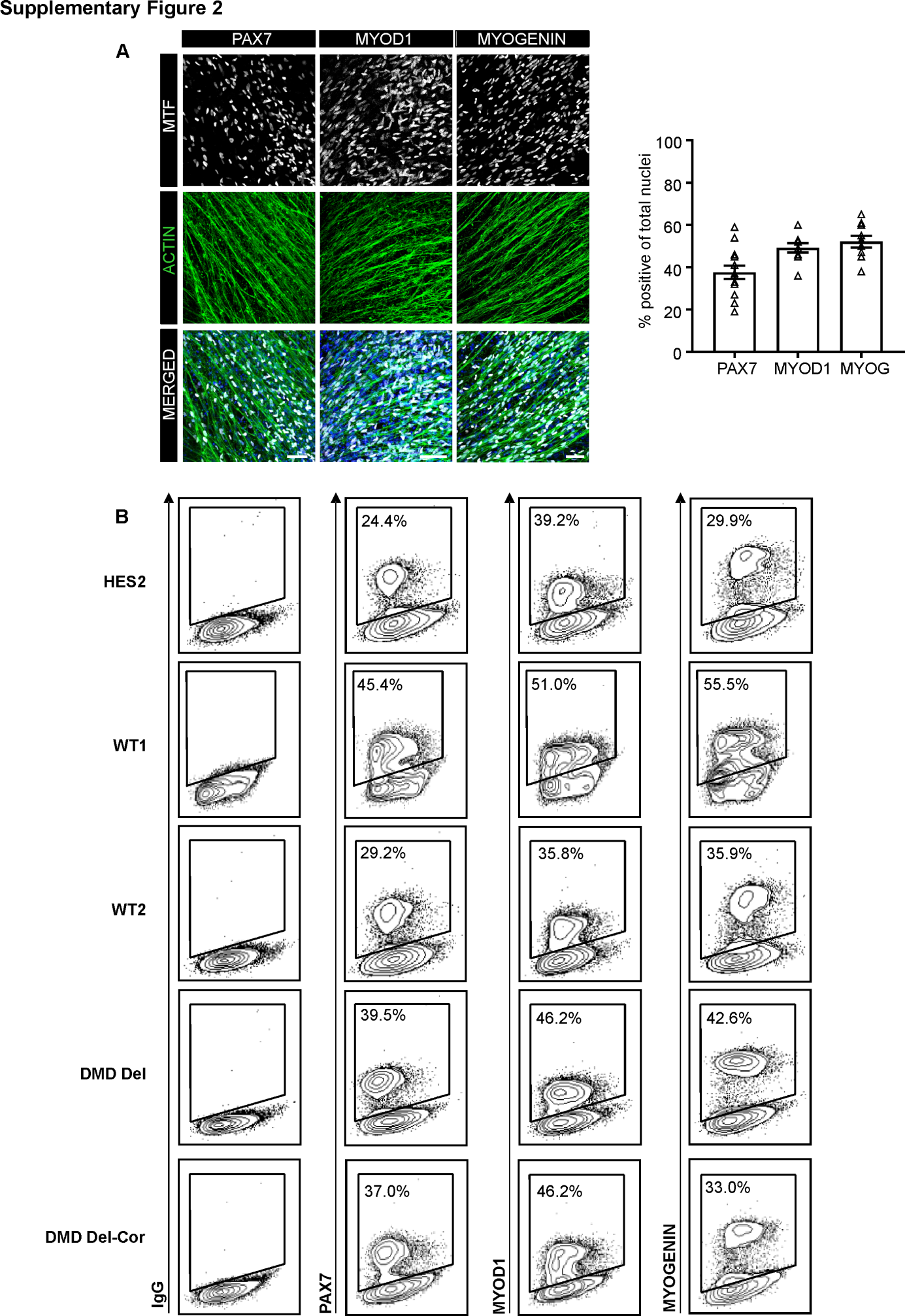


**Supplementary Figure 2. Efficiency of skeletal myocyte differentiation from human PSC.** (**A**) Representative immunostaining of myogenic transcription factors: PAX7, MYOD1 and MYOGENIN (gray), actin (green) and nuclei (blue) in 22 days old skeletal muscle cultures from TC1133 (WT 1) line; Scale bars: 50 µm. Quantification of nuclei positive for PAX7, MYOD1 and MYOGENIN in 22 days old myogenic cultures from HES2 and from iPSC (WT 1) lines; n = 9 -13 differentiations. (**B**) Flow cytometry of myogenic transcription factors PAX7, MYOD1, MYOGENIN in comparison to isotype control (IgG) in day 22 old skeletal myocyte cultures from one ESC line (HES2) and four iPSC lines (WT1, WT2, DMD Del, DMD Del-Cor).

**
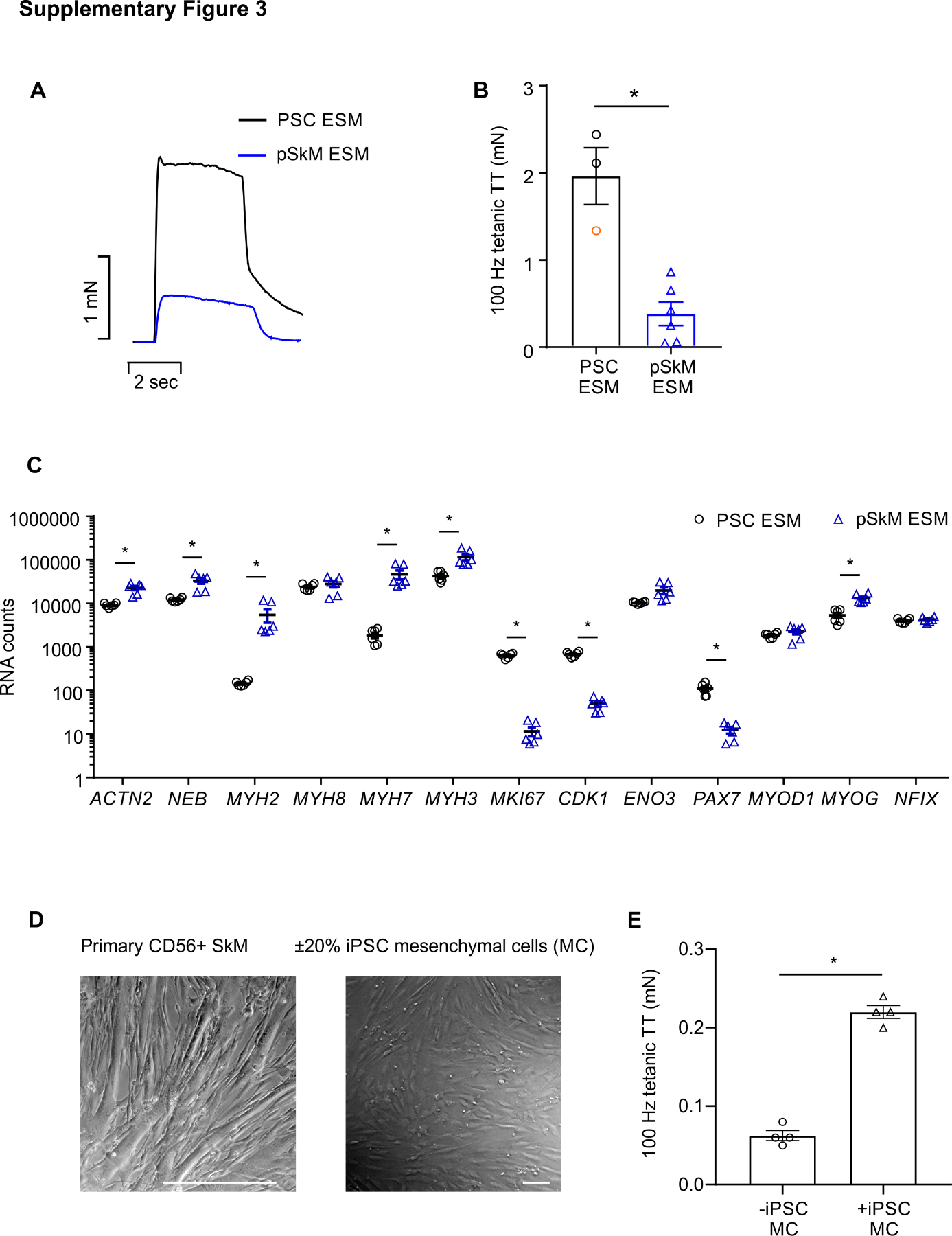
**

**Supplementary Figure 3. Comparison of ESM from PSC and primary skeletal myocytes.** **(A)** Representative original twitch tension traces at 100 Hz tetanic contraction of ESM prepared from PSC-derived (PSC ESM) or biopsy-derived primary SkM (pSkM ESM). **(B)** 100 Hz tetanic twitch tension (TT) of ESM from HES2 (orange circle) and WT1, WT2 iPSC lines (black circles), and ESM from 6 different patient biopsies (pSkM ESM); *p<0.05 by unpaired, two-tailed Student’s t-test. **(C)** Transcript levels (RNA counts measured by nCounter) of indicated muscle genes in PSC ESM or pSkM ESM, n = 6, p<0.05 by 2-way ANOVA and Sidak’s multiple comparison test. **(D)** Brightfield images of primary skeletal myoblast/myotube cultures after magnetic cell sorting for CD56 and mesenchymal cells from skeletal muscle differentiation after magnetic cell sorting with anti-Fibroblast beads. Scale bar: 100 µm**. (E)** 100 Hz tetanic twitch tension (TT) of ESM generated without or with addition of 20% PSC-derived mesenchymal cells (MC), n = 4, *p<0.05 by Student’s t-test.

**
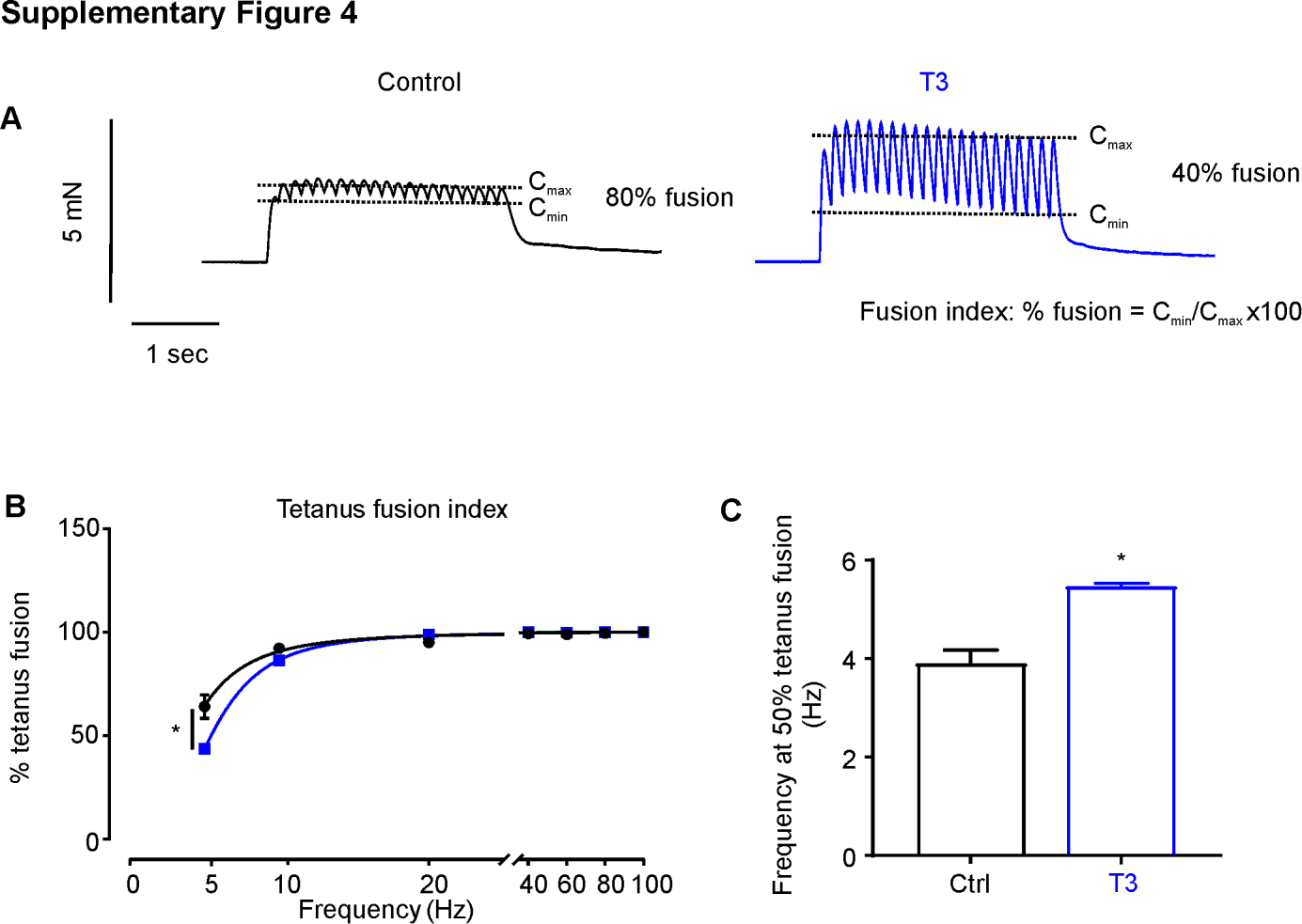
**

Supplementary Figure 4. Thyroid hormone elevates the tetanus threshold of ESM. (A) Fusion index calculated on representative traces of twitch tension generated by control (black) and +T3 (blue) ESM (9 wks old) at 5 Hz tetanus stimulation. The fusion index calculated as the percentage ratio of the maximal relaxation amplitude before the last contraction of the tetanus (C_min_) to the amplitude of this last contraction (C_max_). (B) The fusion index-frequency curve of control (black line) and +T3 (blue line) ESM. *p<0.05 by 2 way-ANOVA and Tukey’s multiple comparison test. (C) Stimulation frequency at 50% tetanus fusion of control (black bar) and +T3 (blue bar) ESM; n = 8/group, *p<0.05 by Student’s t-test.

**
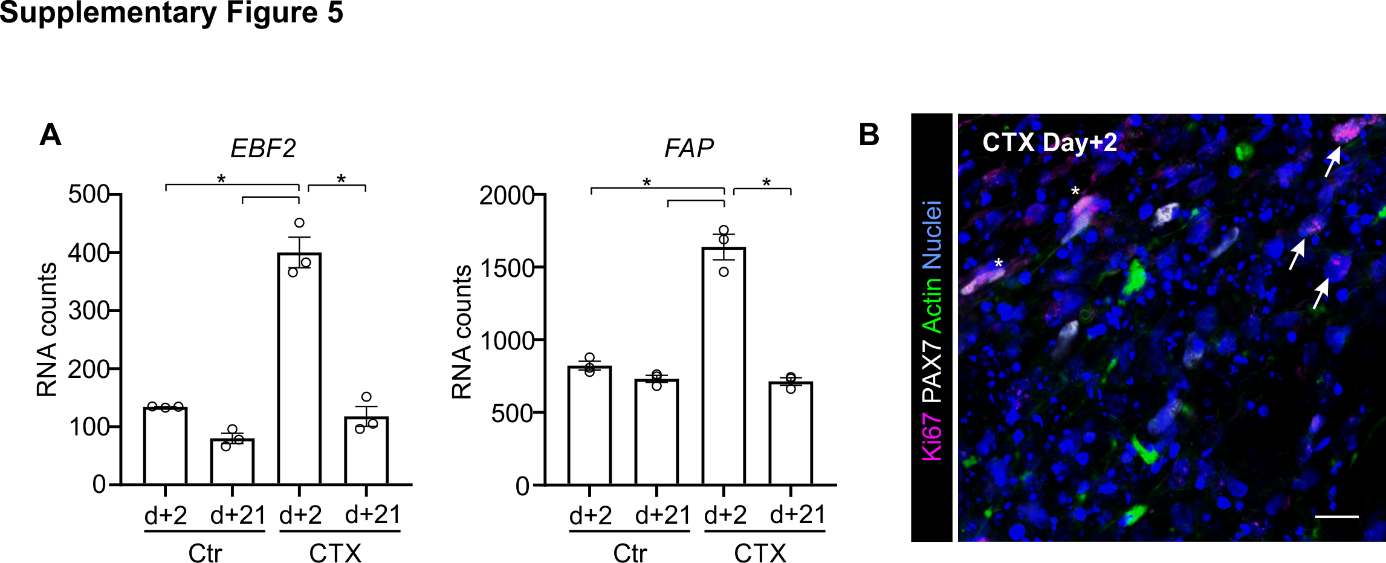
**

**Supplementary Figure 5. Activation of mesenchymal cells by CTX injury. (A)** RNA transcript abundance for indicated non-myocyte genes at early (d+2) and late (day+21) time points after CTX (25 µg/ml) injury or control (Ctrl) conditions; n=3, *p<0.05 by 1-way ANOVA and Tukey’s multiple comparison test. **(B)** Immunostaining of injured ESM for Ki67 (magenta), actin (green), PAX7 (gray), and nuclei (blue). Arrows label Ki67^+^ PAX7^-^ cells, stars label Ki67^+^ PAX7^+^ cells; Scale bar: 20 µm.

**
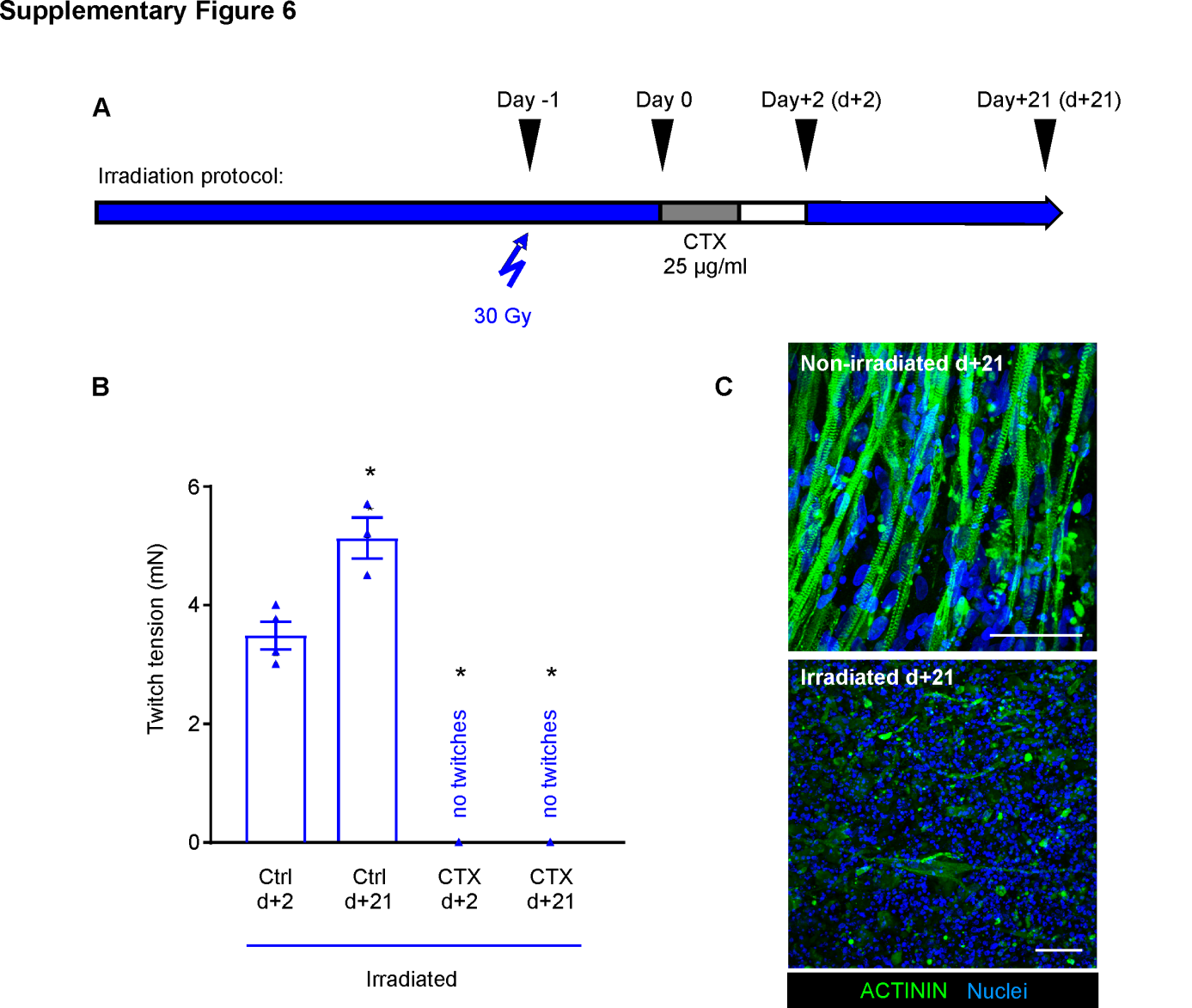
**

**Supplementary Figure 6.** **Irradiation blocks regenerative capacity of human engineered skeletal muscle.** **(A)** Experimental scheme of irradiation protocol. One day before cardiotoxin (CTX) injury ESM were irradiated with 30 Gy. ESM were then incubated with 25 µg/ml CTX for 24 hrs. **(B)** Tetanic twitch tension at 100 Hz stimulation frequency of ESM with irradiation at indicated time points after CTX (25 µg/ml) injury or control (Ctrl) condition; n=3-4/group, *p<0.05 vs. Ctrl day+2 by 1-way ANOVA and Tukey’s multiple comparison test. **(C)** Immunostaining of sarcomeric α-actinin (green) and nuclei (blue) in non-irradiated ESM **(top panel)** and irradiated ESM **(bottom panel)** 21 days after CTX injury. Scale bars: 50 µm.

**
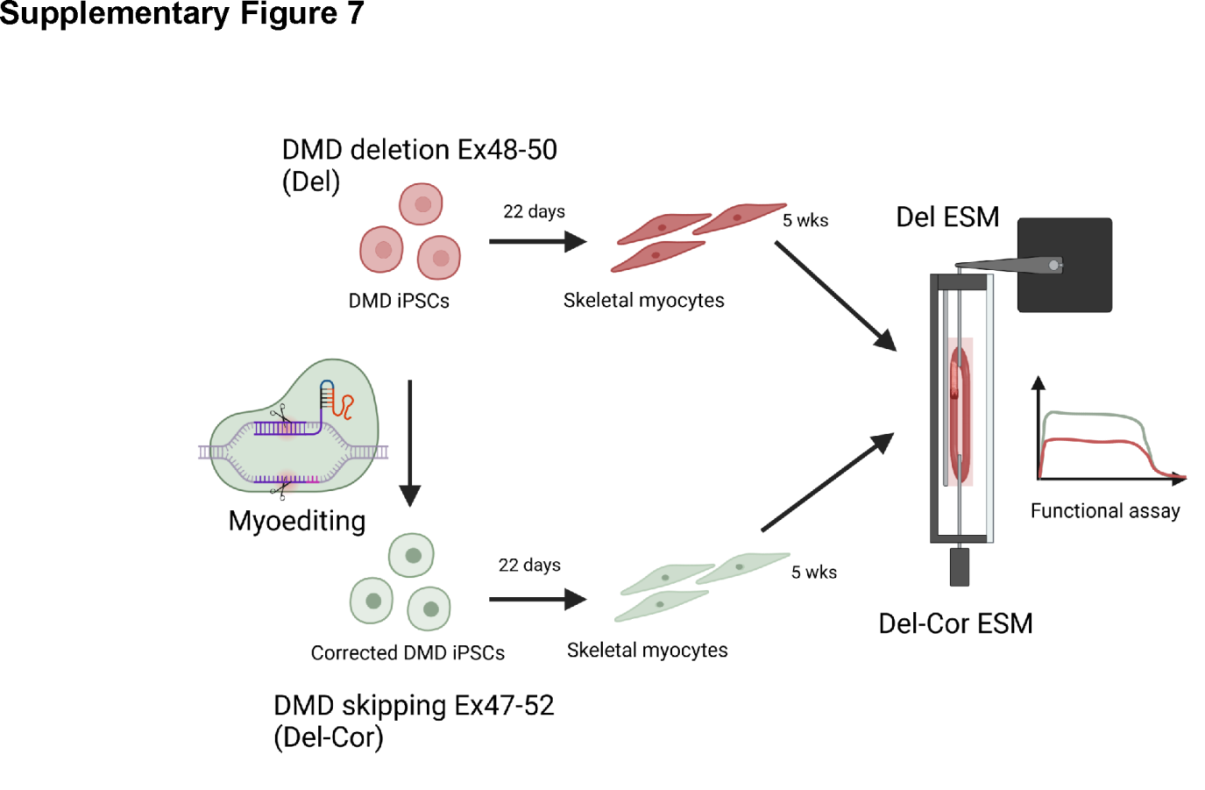
**

**Supplementary Figure 7. Modeling Duchenne muscular dystrophy in ESM.** Scheme of experimental setup.

**Supplementary videos**

**Video 1: Spontaneous contractions 2D skeletal myocyte cultures (day 30)**

**Video 2: Spontaneous contractions of SMO matured for 3 weeks on metal holders**

**Video 3: Spontaneous contractions of ESM matured for 2 weeks on metal holders**

**Supplementary references**

1. Baghbaderani BA, Tian X, Neo BH, Burkall A, Dimezzo T, Sierra G, et al. cGMP-Manufactured Human Induced Pluripotent Stem Cells Are Available for Pre-clinical and Clinical Applications. Stem Cell Reports. 2015;5:647-59.

2. Kamdar F, Das S, Gong W, Klaassen Kamdar A, Meyers TA, Shah P, et al. Stem Cell-Derived Cardiomyocytes and Beta-Adrenergic Receptor Blockade in Duchenne Muscular Dystrophy Cardiomyopathy. J Am Coll Cardiol. 2020;75:1159-74.

3. Darabi R, Arpke RW, Irion S, Dimos JT, Grskovic M, Kyba M, et al. Human ES- and iPS-derived myogenic progenitors restore DYSTROPHIN and improve contractility upon transplantation in dystrophic mice. Cell Stem Cell. 2012;10:610-9.

4. Long C, Li H, Tiburcy M, Rodriguez-Caycedo C, Kyrychenko V, Zhou H, et al. Correction of diverse muscular dystrophy mutations in human engineered heart muscle by single-site genome editing. Sci Adv. 2018;4:eaap9004.

5. Schmidt J, Barthel K, Wrede A, Salajegheh M, Bahr M, Dalakas MC. Interrelation of inflammation and APP in sIBM: IL-1 beta induces accumulation of beta-amyloid in skeletal muscle. Brain. 2008;131:1228-40.

6. Soong PL, Tiburcy M, Zimmermann WH. Cardiac differentiation of human embryonic stem cells and their assembly into engineered heart muscle. Curr Protoc Cell Biol. 2012;Chapter 23:Unit23 8.

7. Tiburcy M, Meyer T, Soong PL, Zimmermann WH. Collagen-based engineered heart muscle. Methods Mol Biol. 2014;1181:167-76.

8. Tiburcy M, Hudson JE, Balfanz P, Schlick S, Meyer T, Chang Liao ML, et al. Defined Engineered Human Myocardium With Advanced Maturation for Applications in Heart Failure Modeling and Repair. Circulation. 2017;135:1832-47.

9. Zimmermann WH, Fink C, Kralisch D, Remmers U, Weil J, Eschenhagen T. Three-dimensional engineered heart tissue from neonatal rat cardiac myocytes. Biotechnol Bioeng. 2000;68:106-14.

10. Tiburcy M, Markov A, Kraemer LK, Christalla P, Rave-Fraenk M, Fischer HJ, et al. Regeneration competent satellite cell niches in rat engineered skeletal muscle. FASEB Bioadv. 2019;1:731-46.

11. Dobin A, Davis CA, Schlesinger F, Drenkow J, Zaleski C, Jha S, et al. STAR: ultrafast universal RNA-seq aligner. Bioinformatics. 2013;29:15-21.

12. Liao Y, Smyth GK, Shi W. featureCounts: an efficient general purpose program for assigning sequence reads to genomic features. Bioinformatics. 2014;30:923-30.

13. Xi H, Fujiwara W, Gonzalez K, Jan M, Liebscher S, Van Handel B, et al. In Vivo Human Somitogenesis Guides Somite Development from hPSCs. Cell Rep. 2017;18:1573-85.

14. Wolf FA, Angerer P, Theis FJ. SCANPY: large-scale single-cell gene expression data analysis. Genome Biol. 2018;19:15.

15. Eisenberg E, Levanon EY. Human housekeeping genes, revisited. Trends Genet. 2013;29:569-74.

16. Becht E, McInnes L, Healy J, Dutertre CA, Kwok IWH, Ng LG, et al. Dimensionality reduction for visualizing single-cell data using UMAP. Nat Biotechnol. 2019;37:38-44
